# Interference with the production of infectious viral particles and bimodal inhibition of replication are broadly conserved antiviral properties of IFITMs

**DOI:** 10.1371/journal.ppat.1006610

**Published:** 2017-09-28

**Authors:** Kevin Tartour, Xuan-Nhi Nguyen, Romain Appourchaux, Sonia Assil, Véronique Barateau, Louis-Marie Bloyet, Julien Burlaud Gaillard, Marie-Pierre Confort, Beatriz Escudero-Perez, Henri Gruffat, Saw See Hong, Marie Moroso, Olivier Reynard, Stéphanie Reynard, Elodie Decembre, Najate Ftaich, Axel Rossi, Nannan Wu, Frédérick Arnaud, Sylvain Baize, Marlène Dreux, Denis Gerlier, Glaucia Paranhos-Baccala, Viktor Volchkov, Philippe Roingeard, Andrea Cimarelli

**Affiliations:** 1 CIRI, Centre International de Recherche en Infectiologie, Lyon, France; 2 INSERM, U1111, Lyon, France; 3 Université Claude Bernard Lyon1, Lyon, France; 4 CNRS, UMR5308, Lyon, France; 5 Ecole Normale Supérieure de Lyon, Lyon, France; 6 Univ Lyon, Lyon, France, Lyon, France; 7 Plateforme IBiSA de Microscopie Electronique, Université F. Rabelais et CHRU de Tours, Tours, France; 8 INSERM U966, Université F. Rabelais et CHRU de Tours, Tours, France; 9 IVPC UMR754, INRA, Univ Lyon, Université Claude Bernard Lyon1, EPHE, Lyon, France; 10 Fondation Mérieux, Lyon, France; 11 Institut Pasteur, Lyon, France; 12 Institute of BioMedical Science (IBMS), East China Normal University (ECNU), Shanghai, China; University of Wisconsin, UNITED STATES

## Abstract

IFITMs are broad antiviral factors that block incoming virions in endosomal vesicles, protecting target cells from infection. In the case of HIV-1, we and others reported the existence of an additional antiviral mechanism through which IFITMs lead to the production of virions of reduced infectivity. However, whether this second mechanism of inhibition is unique to HIV or extends to other viruses is currently unknown. To address this question, we have analyzed the susceptibility of a broad spectrum of viruses to the negative imprinting of the virion particles infectivity by IFITMs. The results we have gathered indicate that this second antiviral property of IFITMs extends well beyond HIV and we were able to identify viruses susceptible to the three IFITMs altogether (HIV-1, SIV, MLV, MPMV, VSV, MeV, EBOV, WNV), as well as viruses that displayed a member-specific susceptibility (EBV, DUGV), or were resistant to all IFITMs (HCV, RVFV, MOPV, AAV). The swapping of genetic elements between resistant and susceptible viruses allowed us to point to specificities in the viral mode of assembly, rather than glycoproteins as dominant factors of susceptibility. However, we also show that, contrarily to X4-, R5-tropic HIV-1 envelopes confer resistance against IFITM3, suggesting that viral receptors add an additional layer of complexity in the IFITMs-HIV interplay. Lastly, we show that the overall antiviral effects ascribed to IFITMs during spreading infections, are the result of a bimodal inhibition in which IFITMs act both by protecting target cells from incoming viruses and in driving the production of virions of reduced infectivity. Overall, our study reports for the first time that the negative imprinting of the virion particles infectivity is a conserved antiviral property of IFITMs and establishes IFITMs as a paradigm of restriction factor capable of interfering with two distinct phases of a virus life cycle.

## Author summary

IFITMs are interferon-regulated proteins that inhibit a broad range of viruses. Until recently, IFITMs had been described to arrest incoming viral particles in target cells, by inducing their retention in endosomal vesicles. More recently in the case of HIV-1, ours and other laboratories have highlighted the existence of an additional antiviral mechanism with which IFITMs could act in virus-producing cells, leading to the production of virion particles of reduced infectivity. In the present study, we assessed whether the negative imprinting of the virion particles infectivity was a conserved antiviral property of IFITMs by examining a panel of fourteen different DNA or RNA viruses. Our results indicate that a wide spectrum of viruses is susceptible to this antiviral mechanism of inhibition, although some are able to resist it. Swapping of elements between susceptible and resistant viruses strongly suggests that specificities in the mode of virion assembly and not the viral glycoprotein are the dominant factor in the susceptibility of a given virus to this inhibition. However, we also show that HIV-1 strains that engage the CCR5 co-receptor display a notable resistance towards IFITM3, indicating that at least in the case of HIV-1, co-receptor usage is likely to add an additional layer of complexity in the relationship established between IFITMs and the virus, that may or may not extend to other viral families as well. In the context of spreading infections, the results of this study highlight that the overall antiviral effect of IFITMs is mechanistically caused by a previously unappreciated dual mode of action in which they act both in target cells and in virus-producing cells, by respectively forcing endosome trapping of incoming viruses and by commandeering the formation of new virion particles of reduced infectivity. Overall, the results presented here indicate that the negative imprinting of viral particles is a largely conserved antiviral feature of IFITMs and point to IFITMs as a novel paradigm of innate defense proteins capable of interfering with viral replication at two distinct steps of a virus life cycle.

## Introduction

The interferon-induced transmembrane proteins (IFITMs) are a family of highly related proteins that present two transmembrane domains (TM) connected by a short linker region and an N and C-termini of variable length [1, 2]. In humans, this family is composed of five expressed members: IFITM1, 2 and 3 that are *bona fide* interferon-regulated genes [3], IFITM5 mainly expressed in bone tissue and genetically linked to *Osteogenesis Imperfecta* [4, 5], and IFITM10 that remains poorly characterized. Following their initial identification as antiviral modulators of Influenza virus infection in a functional genomic screen [6], several studies have concurred in establishing IFITM1, 2 and 3 (hereafter referred to as IFITMs) as key components of cellular innate defenses with large antiviral spectrum (acting against Influenza virus, Filoviruses, Coronaviruses, HIV etc. [6–15, 16, 17, 18, 19, 20, 21, 22–28] [29–33]

The first and most studied mechanism through which IFITMs interfere with viral replication takes place in target cells. In this setting, IFITMs sequester incoming virion particles in endosomes by preventing viral-to-cellular membrane fusion, a phenomenon that first impedes the access of the virus to the cell cytoplasm and subsequently leads to its degradation [6, 8, 9, 12, 14, 16, 20, 23–25, 34–39]. It is interesting to note that this mechanism of inhibition, albeit with efficacies that depend on both the IFITM and the virus considered, targets viruses for which the passage through the acidic pH of endosomes is mandatory to trigger viral-to-cellular membranes fusion, as well as those for which this passage is not obligatory, as HIV-1 [23, 40, 41, 42].

While all studies concur in indicating a strong effect of IFITMs on the fusogenic properties of membranes, the precise phase affected by IFITMs remains debated, given that IFITMs have been described to block hemifusion (the process whereby the outer, but not the inner, leaflet of the viral and cellular membranes merge [35], or the transition from hemifusion to pore formation [43]). Similarly, the exact molecular mechanism by which IFITMs modify the biophysical properties of membranes remains unclear, having been controversially linked in the past to changes in membrane cholesterol levels [44, 45], or more recently to interactions with other cellular co-factors [46].

In the case of HIV-1, our laboratory together with others has recently described an additional mechanism through which IFITMs coalesce with the HIV-1 structural protein Gag during virion assembly and are incorporated into newly-produced particles that display a reduced infectivity when compared to their WT counterparts, due to a decreased ability to fuse with cellular membranes [18, 19, 22]. This novel antiviral effect that we refer to as a negative imprinting of the viral particle infectivity is independent of the cell type in which viruses are produced, as it is observed in established cell lines and in primary cell targets of HIV-1 replication, such as T cells [19, 22] and macrophages [18]. Altogether, these data along with data in the literature suggests that, at least in the case of HIV-1, IFITMs bears the ability to inhibit the virus life cycle at two distinct moments: i) during viral entry in target cells, step in which IFITMs intervene by trapping incoming virions in endosomal vesicles; ii) during virion particle assembly, phase in which the presence of IFITMs leads to the production of virions of reduced infectivity.

However, although the former antiviral property of IFITMs has been firmly established to target a broad range of viruses, it is entirely unappreciated whether the second is restricted to HIV or whether it applies to other viruses.

To address this question, we examined fourteen different viruses, encompassing a wide spectrum of viral families with diverse modes of replication, and in particular: negative-strand RNA viruses (Vesicular Stomatitis virus, VSV; Measles virus, MeV; Ebolavirus, EBOV; Dugbe virus, DUGV; Rift Valley fever virus, RVFV and Mopeia virus, MOPV); positive-strand RNA viruses (West Nile virus, WNV and Hepatitis C virus, HCV); retroviruses (Simian Immunodeficiency virus of macaques, SIV_MAC_; Murine Leukemia virus, MLV; Mason-Pfizer monkey virus, MPMV; in comparison to the already described HIV-1); and DNA viruses (Epstein-Barr virus, EBV; and Adeno-associated virus, AAV).

For the first time, we show here that IFITMs reduce the infectivity of newly produced virion particles derived from a large spectrum of viruses, well beyond HIV-1. In addition, we highlight viruses that are either fully resistant to IFITMs (HCV, RVFV, MOPV along with AAV), or that display an exquisite IFITM member-specific susceptibility (DUGV, EBV). This heterogeneous behavior allowed us to use genetic elements swapping between IFITM-resistant and –susceptible viruses and to point to the specificities in the mode of virion assembly rather than to the viral glycoprotein as a dominant factor of susceptibility towards IFITMs. However, in agreement with recent studies in the literature [23, 47, 48], we also show that, contrarily to X4-tropic, R5-tropic HIV-1 strains become resistant to IFITM3, likely underlying a novel role for co-receptors in the interplay between HIV and IFITMs. Finally, we have re-examined the effects played by IFITMs during spreading infections and we now reveal that they are mechanistically due to the concurrent action exerted by IFITMs both in target cells protection and in the production of virions of reduced infectivity. To our knowledge, this provides the first example of antiviral factor capable of targeting both extremes of a virus life cycle.

## Results

### Evaluating the effects of the expression of IFITMs on the production of virion particles derived from a broad panel of viruses

**Fig 1:**
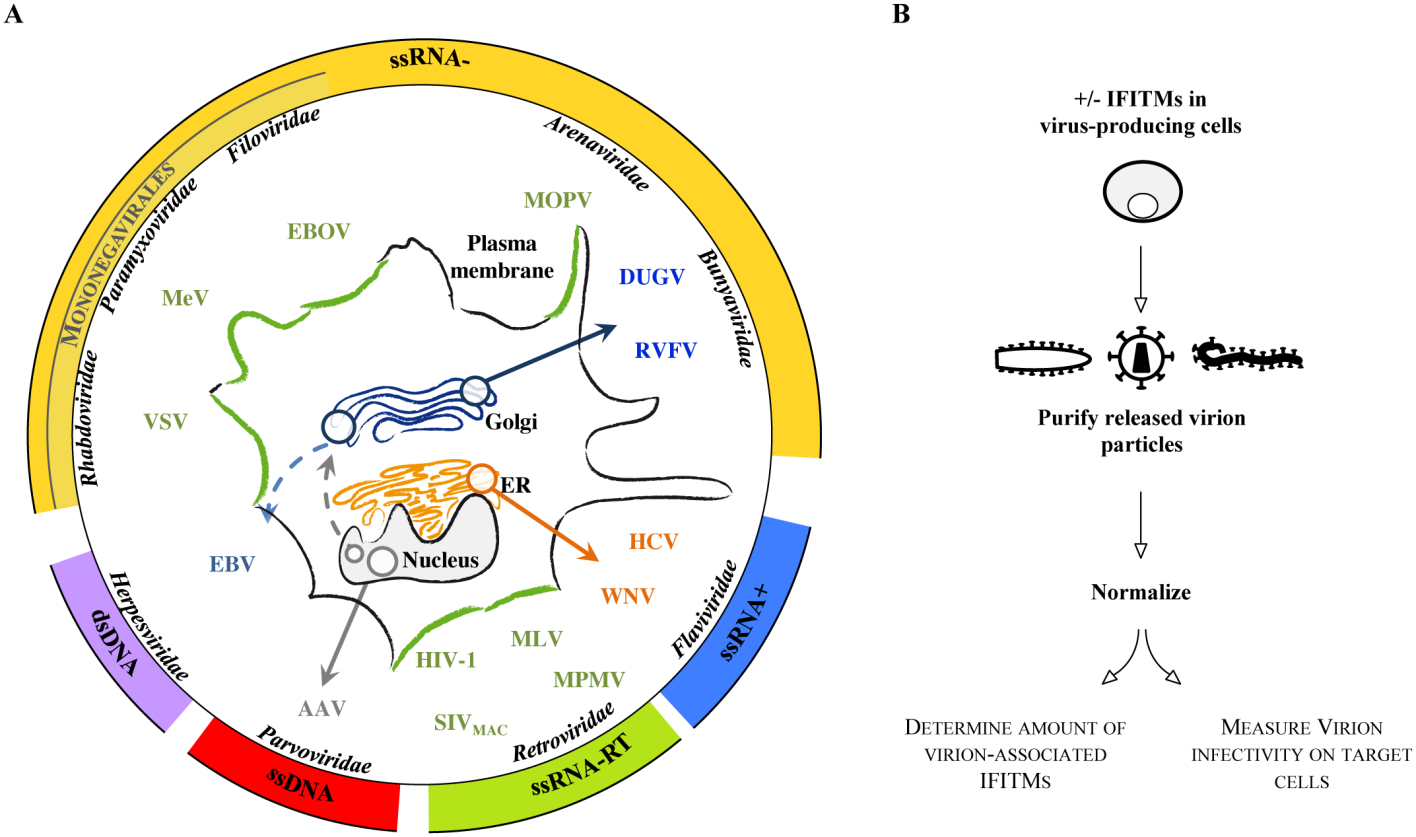
Experimental overview of the present study. Snapshot presentation of the viruses used in this study and schematic color-coded representation of the intracellular locations of virion particle assembly (A). Experimental setup used to determine effects of IFITMs on the formation of infectious viral particles (B). A comprehensive table of the viral systems used is provided in Supplementary S1 Fig.

To assess the degree of conservation of the IFITM-mediated negative imprinting on the virion particles infectivity, we examined a panel of fourteen viruses spanning families distinct in terms of genome, replication, interaction with their cellular host and more interestingly, presenting different modes of virion assembly (as schematically presented in Fig 1A). This list included several members of the *Retroviridae* family and in particular MLV (genus *Gammaretrovirus*), MPMV (genus *Betaretrovirus*) as well as SIV_MAC_ and HIV-1 (genus *Lentivirus*, the latter of which we had previously characterized with respect to its susceptibility to IFITMs, [18]); the plus-strand RNA *Flaviviridae* WNV (genus *Flavivirus*) and HCV (genus *Hepacivirus*); representative members of different minus-strand RNA virus families such as *Rhabdoviridae* (VSV), *Paramyxoviridae* (MeV); *Filoviridae* (EBOV), as well as segmented RNA viruses belonging to *Bunyaviridae* (DUGV of the genus *Nairovirus* and RVFV, of the genus *Phlebovirus*) and *Arenaviridae* (MOPV). To complete this analysis, two DNA viruses respectively members of the *Herpesviridae* (EBV) and *Parvoviridae* families (AAV) were also included here. Contrarily to all the viruses examined in this study, AAV is a non-enveloped virus.

To minimize the inevitable variations dictated by the specificities of each one of these viruses (cellular tropism, availability of cloned genomes, of viral-coded reporters and so forth), we decided to use an as much as possible homogenous setup to assess the ability of IFITM1, 2 or 3 to interfere with the production of infectious virions (as schematically presented in Fig 1B and as more specifically detailed in Supplementary S1 Fig).

IFITMs are expressed in virus-producing cells in conditions and amounts that are comparable to those that can be measured in primary monocyte-derived dendritic cells (MDDCs) stimulated with type I IFN (Supplementary S2A, S2B, S2C and S2D Fig) and that we had previously employed to reveal this novel antiviral mechanism in the context of HIV-1 [18]. Due to cell type specific transfection rates, the intracellular levels of IFITMs measured upon transient DNA transfection varied among cell lines, although variations were contained within four-fold in terms of both number of IFITM-positive cells and median fluorescent intensity (MFI, as measured by flow cytometry, Supplementary S2A and S2B Fig).

When cloned viral genomes that allow efficient viral rescue upon DNA transfection were available, virion particles were produced by simple co-transfection of viral components along with DNA coding IFITMs (as for HIV-1, SIV_MAC_, MLV, MPMV, WNV and AAV). Otherwise, cells transfected with the individual IFITMs were infected shortly after transfection to obtain a consistent pool of virus-producing IFITMs-expressing cells. Then, viral particles newly-produced in cell free supernatants were isolated and purified by ultracentrifugation through a 25% sucrose cushion (w/v) and then normalized for protein or genome content (Supplemental S1 Fig). Normalized amounts of virion particles produced in the presence or absence of IFITMs were then analyzed by WB or used to challenge target cells to measure their infectivity and possible effects of IFITMs on this parameter (Supplementary S1, S3A and S3B Figs for a comprehensive comparison of the infectious titers used).

Few modifications were used to adapt to specific virus features. Hone cells that bear a latent EBV genome carrying *gfp* were transfected with IFITMs coding DNA along with an expression vector coding the EB1 viral transcription factor to stimulate viral reactivation and virion assembly [49, 50]. Given its assembly in the nucleus, AAV virions were purified from cell lysates using a well-established procedure that includes freeze-thaw cycles and virion purification on a 4 step gradient of iodixanol [51]. For HCV, IFITMs were expressed in hepatocytic Huh-7.5.1c2 cells [52] and to circumvent the poor transfection rate of these cells, expression of IFITMs was obtained by retroviral-mediated transduction.

Finally, while parental viruses were examined in most cases, vectors were used for retroviruses. This allowed us to pseudotype retroviral particles with the same heterologous envelope (the VSV G protein, VSVg) to better appreciate potentially subtler differences due to distinct virion assembly mode existing between *Retroviridae*. VSV-G pseudotyping is commonly used in studies on retroviruses and we had previously determined that IFITMs exert similar defects on the infectivity of HIV-1 viral particles bearing the HIV-1, the VSV-G, the gibbon ape leukemia virus (GALV) or the feline leukemia virus RD114 envelope proteins [18].

When the conditions described above were used, the presence of IFITMs in virus-producing cells did not appreciably modify the amount of virion particles produced, although a trend toward increased viral production was observed for AAV, EBV and MOPV in the presence of IFITM3 and a negative one was observed for HIV-1, MLV and HCV (Supplementary S3C Fig). To focus only on their intrinsic infectivity, all the subsequent assays were performed for each virus after normalization of virion particles produced in the presence or absence of IFITMs.

**Fig 2:**
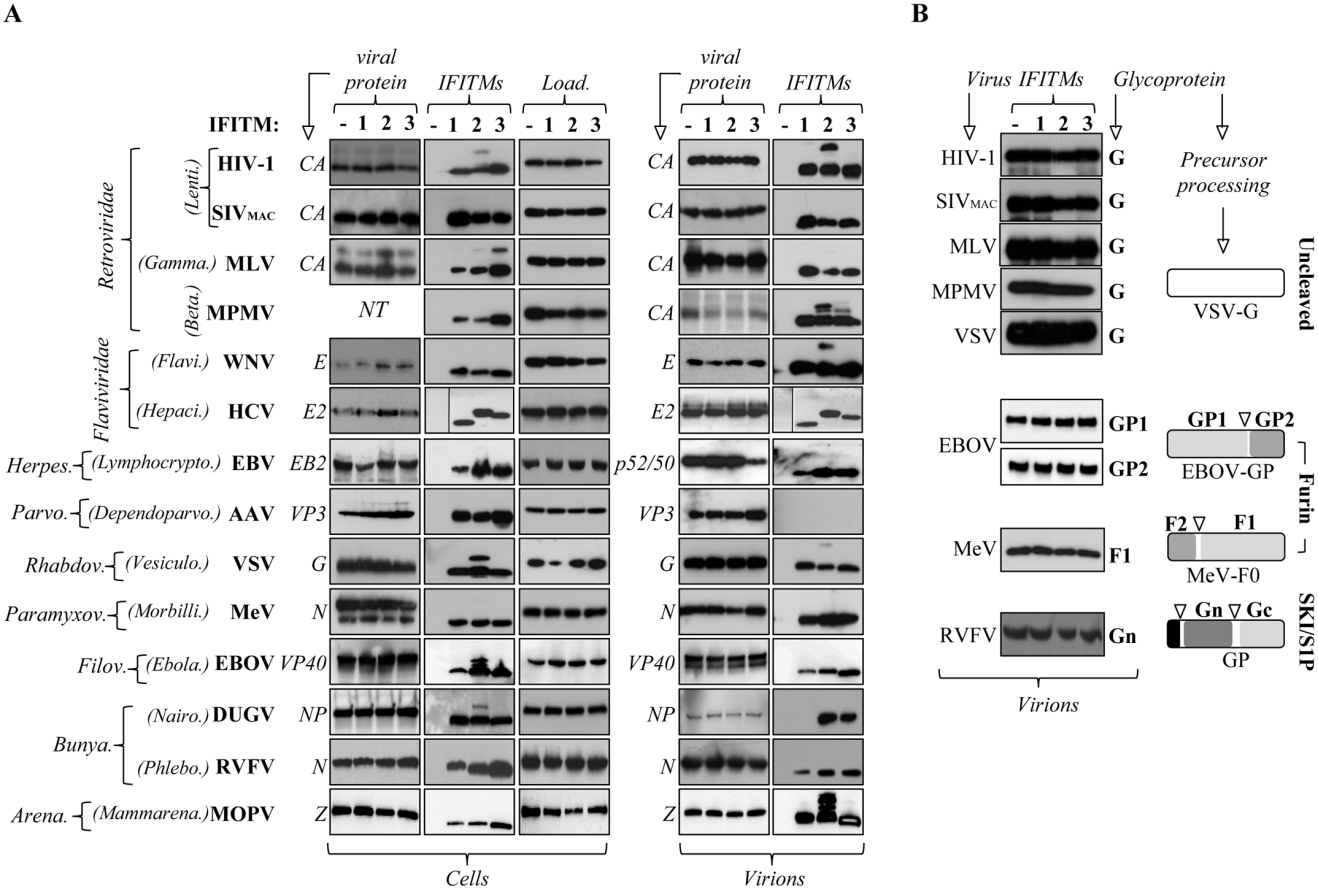
IFITMs associate to viral particles of most, albeit not all, viruses tested and do not induce detectable changes in mature glycoproteins incorporation. A) Virion particles produced in presence or absence of IFITMs and their corresponding cellular lysates were analyzed by WB. Virions were harvested from the cell supernatant and purified by ultracentrifugation through sucrose, with the exception of AAV for which cells were first lysed by freeze/thaw and then virions were purified by ultracentrifugation on a four-step iodixanol gradient. Load.; loading controls using either anti-actin, -tubulin or -EF1α antibodies. Virus-specific antibodies were used according to their availability. In the case of MPMV for which antibodies were not available, CA was identified by comparison with control supernatants and quantified after *Coomassie* staining and densitometry. The panels display representative results of 3 to 5 independent experiments. B) When available, the same viral preparations displayed in A were probed with antibodies specific to the indicated mature glycoproteins. The panels present representative results. The intracellular processing pathways of the viral glycoproteins analyzed is shown schematically at the right.

Next, the ability of IFITMs to be incorporated (or to co-fractionate) in normalized viral preparations was assessed by Western blot (Fig 2A). In line with its non-enveloped characteristics, viral preparations of AAV were devoid of IFITMs even when four times more starting material than the one used for the other viruses was used (Fig 2A). On the contrary, readily detectable amounts of IFITM1, 2 and 3 were observed in viral fractions of all enveloped viruses tested, under the experimental conditions used here. The unique exception was represented by DUGV in which IFITM1 was undetectable in viral preparations, contrarily to IFITM2 and IFITM3 and despite comparable intracellular expression levels. These results are not due to lower viral inputs, because similar amounts of physical DUGV particles were analyzed than other viruses (as for example MOPV and RVFV, Supplementary S3A and S3B Fig).

Overall, the data presented here on fourteen different viruses indicates that IFITMs co-fractionate with most enveloped virion particles in a robust manner.

A direct interaction between the HIV-1 Env and IFITMs has been hypothesized to drive functional alterations of the amount of Envelope spikes present in virion particles [22], although this finding has not been confirmed by other studies, including our own [18, 23, 53]. Given these discrepancies, it was important to determine whether IFITMs could modify or not the extent of mature glycoproteins incorporation in the case of other viruses. To this end, we examined the extent of mature glycoproteins incorporation in virion particles derived from viruses for which antibodies were available (Fig 2B). No specific defect in mature glycoproteins incorporation into virions was observed in the presence of IFITMs, irrespectively of the glycoprotein intracellular processing/maturation pathway (schematically displayed at the right of the panels of Fig 2B), refuting the hypothesis of a generalized mechanism through which IFITMs may interact and/or modify viral glycoproteins maturation and incorporation into virions.

Two further considerations can also be drawn from the comparison of the migration patterns observed for the different IFITMs after SDS-PAGE gel analysis. First, the IFITM1, 2 and 3 proteins in Huh-7.5.1c2 cells (HCV panels) migrate at distinct apparent molecular weights, contrarily to the IFITMs expressed in the other cell types. The reasons for these differences are unclear although both patterns are equally present in the literature and both have been indistinctly linked to an antiviral phenotype [6, 8, 18, 19, 25, 45, 54]. Second, slower migrating forms of IFITMs, especially of IFITM2, can be in some cases observed in cellular and viral fractions. These bands have been observed before, but at present their identity is unknown, although we speculate they may represent post translational modifications of IFITMs. The finding that such forms appear more concentrated in viral preparations of some viruses may suggest their selective enrichment at the sites of virion assembly, an hypothesis that will require further studies.

### Immuno-gold electron microscopy underscores *bona fide* virion-association of IFITMs

**Fig 3:**
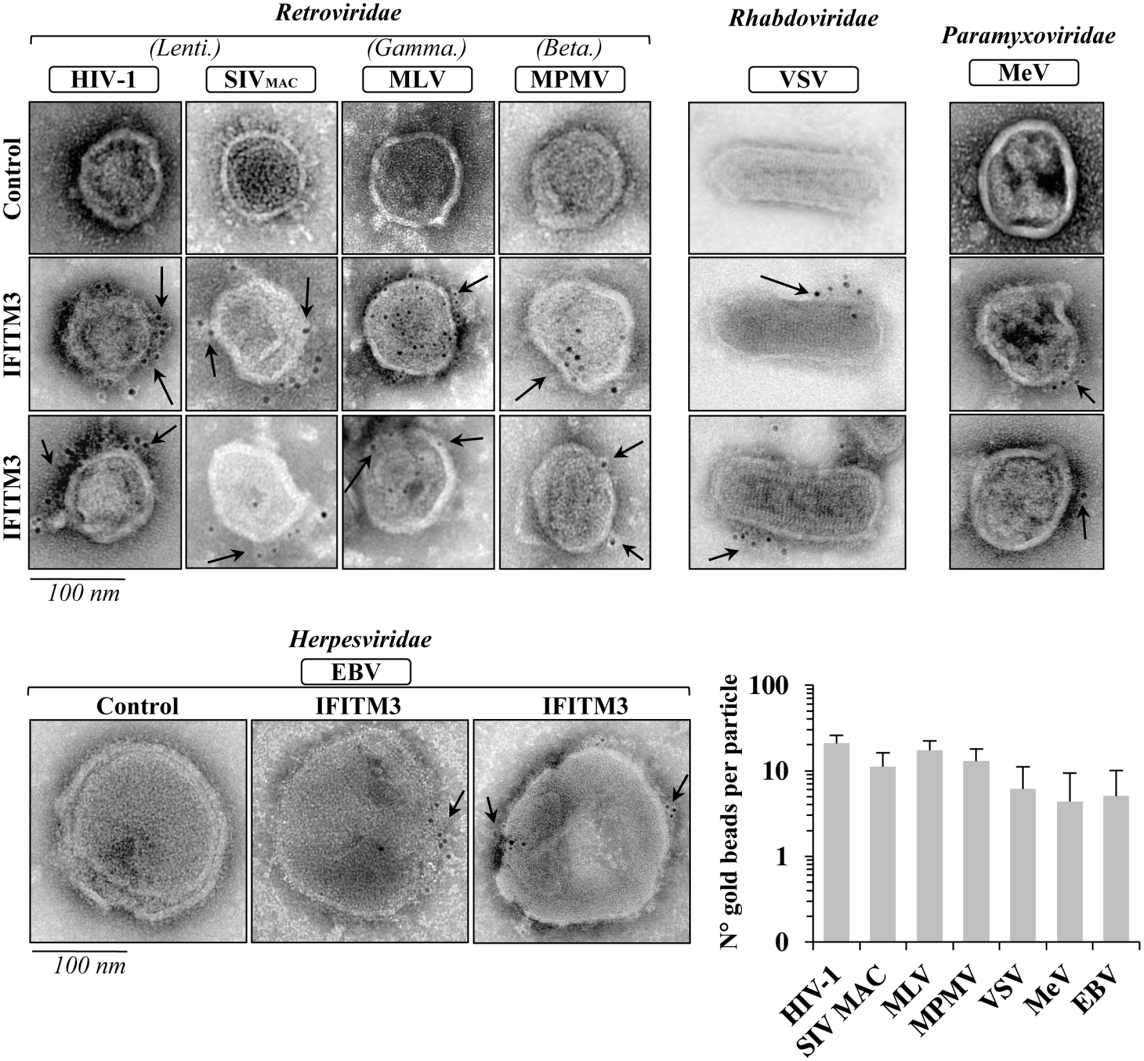
IFITM3 is a *bona fide* virion-associated protein. Virion particles produced as described above were then analyzed by immuno-gold electron microscopy. Briefly, unfixed viral preparations purified by ultracentrifugation and produced in the presence or absence of IFITM3 were incubated with anti-Flag antibodies, followed by incubation with a gold-conjugated secondary antibody (arrows). Representative pictures are shown here. The graph displays the number of gold particles counted on a per virion basis.

To confirm the virion association of IFITMs, several viruses were produced in the presence or absence of IFITM3 and were then subjected to immuno-gold EM using antibodies specific for its N-terminal Flag tag. Virion labeling was conducted on fresh viral preparations, prior to fixation, as these conditions yielded more reproducible results in our hands. Under these conditions, IFITM3 was detectable in all virions tested (HIV-1, SIV_MAC_, MLV, MPMV, VSV, MeV and EBV, Fig 3A and 3B for a quantitative analysis of the number of gold beads per virion particle). Due to technical limitations (BSL3/BSL4 constraints and/or limited availability of viral material), this analysis could not be carried out on the remaining viruses.

Overall, our data showing association of IFITM3 with capsid-containing virion particles derived from different viruses clearly support the notion that IFITMs are indeed incorporated in virions, although we cannot exclude the possibility that a portion of virion-associated IFITMs may derive from non-virion associated material [55].

### Evaluation of the infectivity of virions produced in the presence of IFITMs

**Fig 4:**
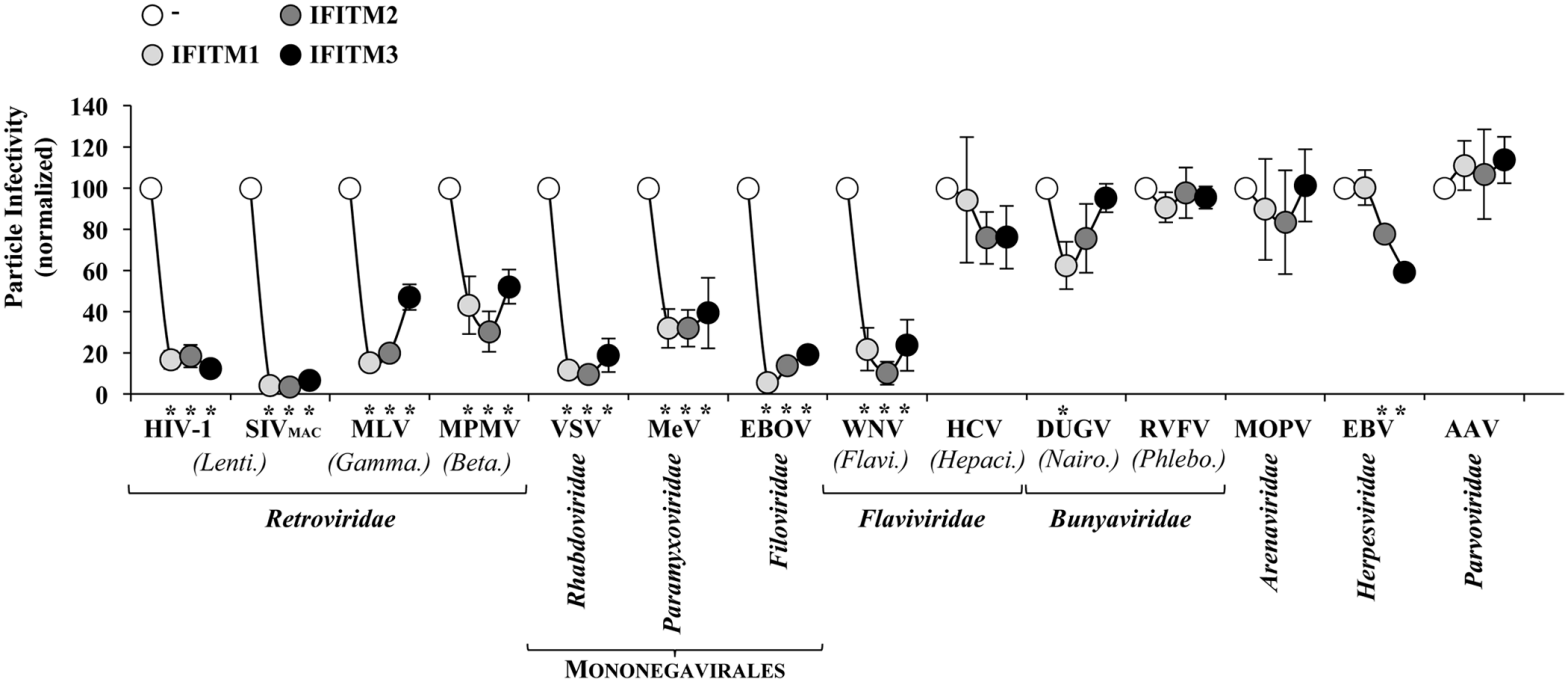
Effect of IFITMs on the production of fully infectious viral particles. Normalized amounts of virion particles produced in the presence of absence of IFITMs were used to challenge target cells prior to analysis of their intrinsic infectivity by FACS, FFA or TCID50, according to the specific virus and as presented in Supplemental S1 Fig. For each virus, infectivity has been normalized to control viruses produced in the absence of IFITMs. The graph presents averages and SEM of 3 to 6 independent experiments. *, statistically significant upon a Student t test, p≤0.05.

Next, the impact of IFITMs on the infectivity of virion particles was determined by infecting cells with normalized amounts of virions (Fig 4). As expected, IFITMs decreased the infectivity of HIV-1 and SIV_MAC_ virion particles. The infectivity of MLV and MPMV was also affected, albeit more moderately in the case of MPMV for all three IFITMs and for MLV specifically for IFITM3 (Fig 4). These results are intriguing because MLV and especially MPMV display assembly specificities that are distinct from lentiviruses, although they ultimately acquire a plasma membrane-derived envelope [56, 57, 58].

Viral infectivity was also significantly impaired in the case of VSV, MeV, EBOV, WNV with no major differences among IFITM members. Instead, DUGV and EBV displayed a member specific susceptibility to IFITMs and in particular, the infectivity of DUGV was impaired to a small but statistically significant extent by IFITM1, but not by IFITM2 or IFITM3, while EBV displayed a reciprocal susceptibility. Lastly, the infectivity of HCV, RVFV, MOPV and AAV virions was unaffected by IFITMs.

N-terminal tagged IFITM proteins are widely used in the field, however it was of importance to ascertain that the presence of the tag was not responsible for the antiviral effects ascribed to IFITMs. To this end, we compared the effects of tagged and non-tagged IFITMs on HIV-1 and VSV, taken here as extremes of viruses susceptible to IFITMs. Under these conditions, both IFITM configurations exerted similar antiviral effects, thereby excluding artefactual effects due to the presence of the tag (Supplementary S4A Fig).

While the behavior of AAV is not surprising given its non-enveloped features, the resistance of HCV, RVFV and MOPV could be in principle due to insufficient levels of expression of IFITMs in virus-producing cells. We do not believe this to be the case, because RVFV, MOPV and AAV were produced in HEK293T cells in conditions similar to Retroviruses and WNV that are instead susceptible to IFITMs (see Supplementary S1 Fig for a comparison of the viral systems used). Similarly, while IFITMs in Huh-7.5.1c2 did not modify the infectivity of HCV particles, they did affect the one of VSV (tested here against IFITM1, Supplementary S4B Fig).

Therefore, we believe that the resistance phenotype described here for HCV, RVFV, MOPV and AAV reflects a true behavior of the virus, rather than limiting amounts of IFITMs in virus-producing cells.

In principle, the lower plasma membrane distribution of IFITM2 and 3 could have been predicted to induce a more moderate infectivity defect for viruses assembling at this location, when compared to IFITM1. However, the cell surface staining of non-permabilized cells (Supplementary S2C and S2D Fig) indicated that IFITM2/3 are clearly present at detectable levels at the plasma membrane, suggesting that membrane dynamics may contribute to flatten differences between individual IFITM members in terms of access to viral assembly sites and antiviral effects.

### CD45-depletion experiments of viral supernatants exclude a confounding role of exosomes in the negative effects of IFITMs on virion infectivity

**Fig 5:**
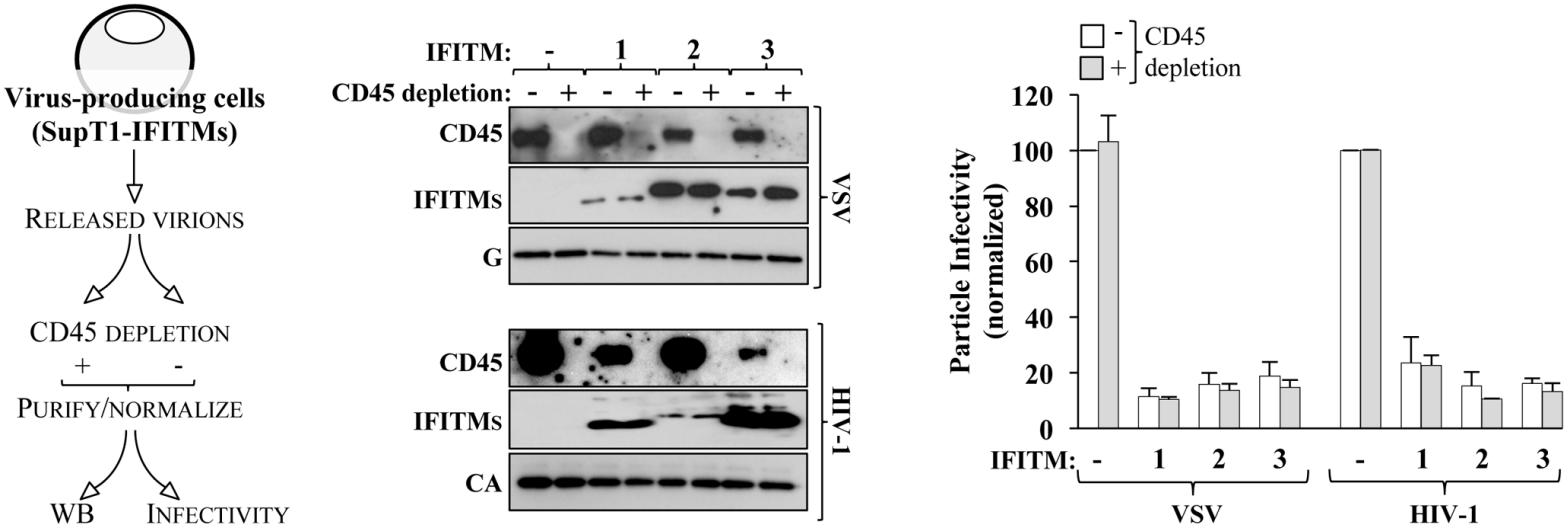
CD45 depletion excludes a potentially confounding role of exosome-incorporated IFITMs on virion infectivity. SupT1 cells stably expressing the different IFITMs were infected with HIV-1 and VSV. At a late time after infection of the cell culture, supernatants containing newly-produced virions were harvested and divided in two fractions that were either incubated with CD45-conjugated microbeads or left untreated. After the microbeads removal, virion particles were purified by ultracentrifugation, normalized and then used for WB and infectivity analyses. The WB panels present typical results obtained, while the graph presents averages and SEM obtained in 3 independent experiments. No statistically significant differences were observed between depleted and non-depleted fractions, after a Student t test.

IFITMs have been shown to be incorporated into virion particles of HIV-1 (and of other viruses in this study), but have also been found to be present in exosomes [55]. To exclude a possible influence of exosomal-associated IFITM proteins in our infectivity assays, we took advantage of a previous study that identified CD45 as a cellular protein specifically excluded from HIV-1 viruses due to a long cytoplasmic tail, but freely present in exosomes [59]. SupT1 cells stably expressing IFITM1, 2 or 3 were infected with HIV-1 and VSV for prolonged periods of time to obtain sufficient amounts of released virion particles produced in the presence of IFITM proteins. Viral preparations were then separated in two and either subjected to CD45 depletion or not. After CD45-magnetic beads removal, virions were purified by ultracentrifugation and upon normalization, they were analyzed by WB and infectivity (Fig 5). CD45 depletion worked efficiently and as our laboratory published before in the case of HIV-1 most of the virion-associated IFITMs signal persisted in the viral fractions, even after CD45 depletion [18]. These results however extend our previous findings indicating that even in the case of VSV, IFITMs become essentially virion-associated in cells undergoing active viral budding. When the infectivity of non-depleted and CD45-depleted virions was analyzed no differences were observed, thereby excluding a possible confounding role of exosomes that may be present in our viral preparations.

### Silencing of endogenous IFITMs increases the basal infectivity of newly-produced virions and augments EBOV spreading

**Fig 6:**
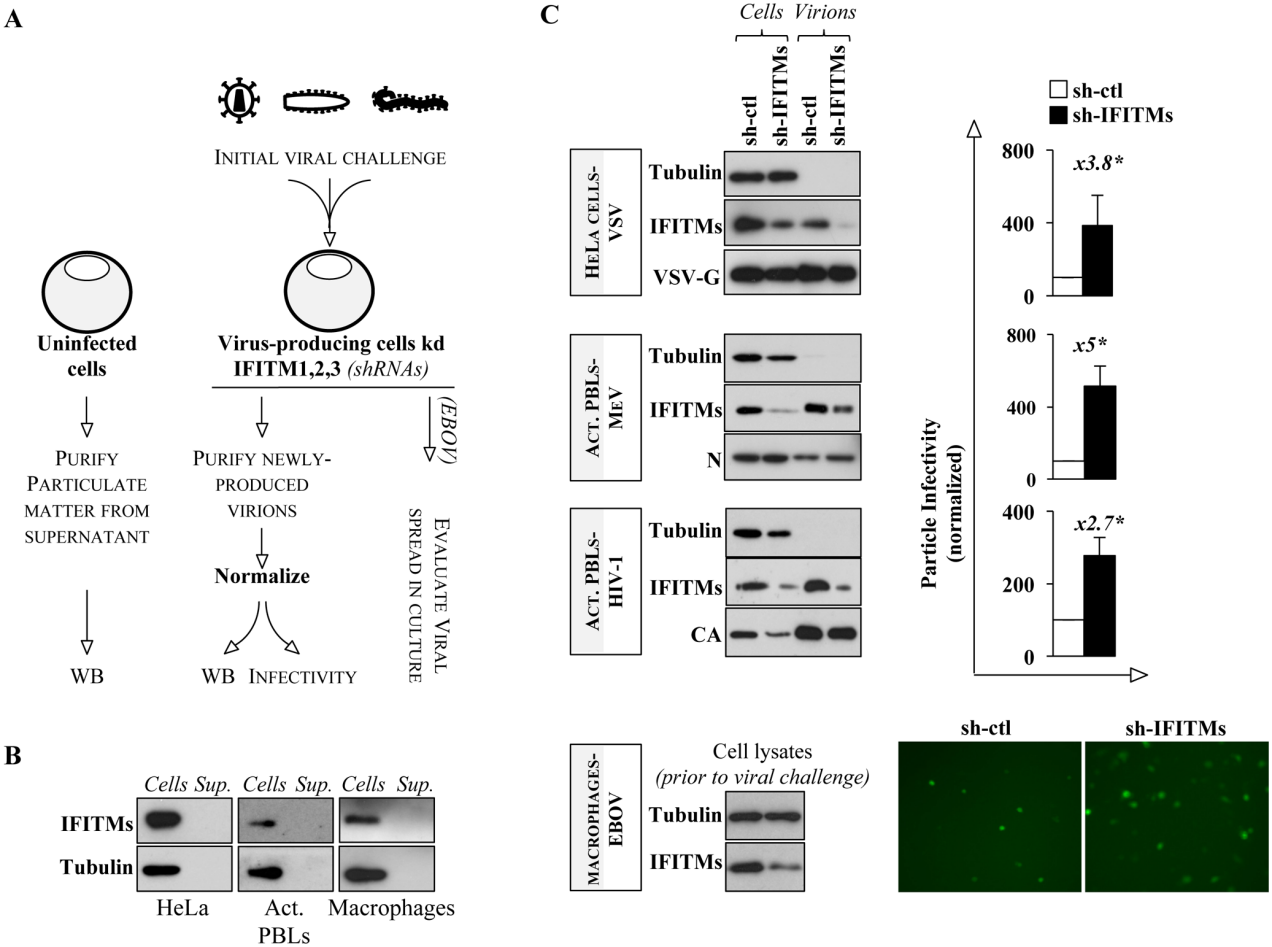
Silencing of endogenous IFITMs increases the infectivity of virions of different viruses and increases EBOV spread in primary macrophages. A) Endogenous IFITM1, 2 and 3 were silenced by shRNA-mediated lentiviral transduction along with shRNA-control silenced cells and then treated according to the scheme provided. B) Expression and extracellular release properties of endogenously-expressed IFITMs in the uninfected cell types used here (cells and sup., respectively). The basal expression levels of IFITM proteins were measured in the cell types mentioned above with a pool of anti-IFITM1, 2 and 3 antibodies in cell lysates and supernatants purified by ultracentrifugation through sucrose, as viral particles in Fig 2. The same amounts of cells used elsewhere for viral production and in the case of primary cells, the same donors were used. C) In the case of primary cells, primary blood lymphocytes were stimulated with 1 μg/ml PHA and 150 U/ml of Interleukin 2 (IL2) for twenty-four hours, then challenged with HIV-1 viral vectors expressing either control (Luciferase), or IFITMs-specific shRNAs and enriched in knockdown cells following a three-day selection in Puromycin (resistance coded by the shRNA vector). Kd-cells were then challenged with the indicated viruses at MOI comprised between 0.1 and 0.5 to obtain virus-producing cells, prior to extensive cell washing to remove input virus. Newly produced virion particles were collected 1 to 2 days afterwards (5 days for HIV-1), purified and normalized prior to WB and infectivity analyses. The infectivity of virions purified from kd-cells was measured on HeLaP4 (HeLa cells bearing the HIV-1 receptors and an LTR-driven promoter driving β-galactosidase expression, used for HIV-1 and VSV and analyzed twenty-four and sixteen hours after challenge by β-gal assay and FACS, respectively) or Vero/hSLAM (MeV, flow cytometry). Primary macrophages were challenged with shRNA-coding vectors in the presence of Vpx-containing virion-like particles (VLPs-Vpx) to increase the efficiency of silencing and then challenged with EBOV at an MOI of 0.3. Pictures of infected cultures were collected with a Leica DM IRB inverted microscope. The graphs present averages and SEM of 4 to 5 independent experiments with cells obtained from different donors. *, p≤0.05 after a Student t test.

To assess the role of endogenous IFITMs, we silenced the three IFITMs at the same time following shRNAs-mediated, lentiviral-based transduction of established cell lines (HeLa cells), or primary blood cells that express them at detectable levels even prior to IFN stimulation (PHA/IL2-activated primary blood lymphocytes, PBLs, and monocyte-derived macrophages). IFN-stimulation was not used to further increase the levels of expression of IFITMs, due to its pleiotropic effects on the cell physiology that would have complicated the analysis of the results obtained. Knockdown cells were then challenged with the indicated viruses and newly-produced virion particles assembled in the absence or presence of endogenous IFITMs were then purified, normalized and their infectivity assessed (according to the scheme provided in Fig 6A). In the case of EBOV infection of primary macrophages silenced for IFITMs, only viral spread could be analyzed due to the limited amount of material available.

In the absence of viral infection, endogenous IFITM proteins were not detected in the supernatant of the cells used here (Fig 6B), further supporting the notion that the major secretion mode for IFITMs under these conditions is their incorporation into virions (as also evident in the WB panels of Fig 6C). As expected, the intracellular reduction of IFITMs, led to a concomitant reduction in the amount of virion-associated IFITMs. When newly-produced virion particles were normalized and used to challenge target cells, the infectivity of virions produced in IFITM-knockdown cells reproducibly increased for all the three viruses tested (VSV, MeV and HIV-1, in this latter case completing the analysis that we had already performed for primary macrophages [18]). Thus, removal of endogenous IFITM proteins results in an increase in the intrinsic infectivity of virion particles, corroborating the results presented in Fig 4. In the case of EBOV, silencing of IFITMs in primary macrophages led to a consistent increase in the replicative capacity of EBOV in the cell culture (Fig 6C displays a single time point of the complete analysis of viral spread over time presented in Supplementary S5 Fig), although the limited material available precluded the purification and the subsequent analysis of the infectivity of newly-produced virions and therefore prevented the assignment, or dismissal, of the antiviral effect of IFITMs on EBOV to a specific mechanism (this issue is addressed in the next figure).

Overall, the data presented here argue for two important points: endogenous IFITMs are indeed incorporated in particles issued from different viruses; their presence leads *de facto* to virion particles of lower infectivity when compared to virion particles produced in their absence.

### The antiviral effects of IFITMs during spreading infections are the result of a bimodal inhibition that operates concomitantly in protecting target cells from infection and in leading to the production of virion particles with reduced infectivity

The data shown above indicates that IFITMs interfere with the infectivity of newly produced virion particles in the same broad manner than their previously reported ability to protect target cells from infection. However, whether the two mechanisms co-exist during spreading infections, and to what extent they contribute to the overall antiviral properties of IFITMs is unknown.

**Fig 7:**
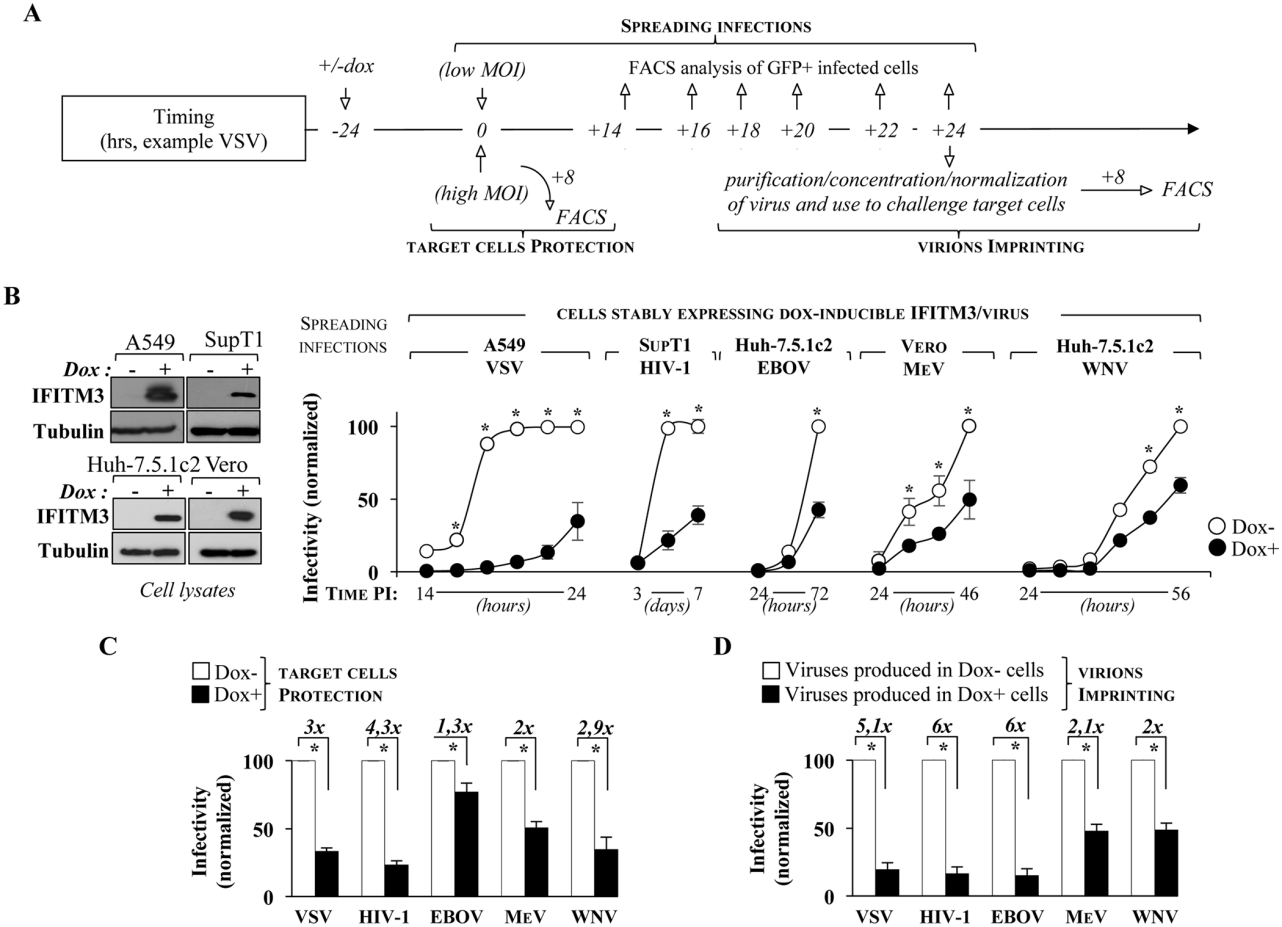
The overall negative effects played by IFITM3 during viral spread are the result of two concomitant antiviral mechanisms, acting in the protection of target cells as well as in the production of virion particles of decreased infectivity. A) Scheme of the experimental approach used here. For simplicity, a detailed time analysis is shown only for VSV. B) Cells stably expressing a dox-inducible IFITM3 were treated or not with doxycycline (for twenty-four hours) and then challenged with the indicated viruses (MOIs comprised between 0.01 and 0.2 depending on the virus). The panels display a typical induction obtained upon twenty-four hours stimulation with concentrations of doxycycline comprised between 1.5 and 10 μg/ml depending on the cell type. The extent of viral spreading through the culture was assessed over time (by FACS since most viruses coded GFP and by exo-RT in the case of HIV-1 that did not). C) The same cells were used to measure the degree of protection offered by IFITM3 towards WT virus infection. To approximate a single round of infection, infections were carried out with high viral inputs (MOIs comprised between 0.1 and 0.5) and the proportion of infected cells was determined shortly after viral challenge by FACS. In the case of HIV-1, single round-competent viruses coding GFP were used to allow flow cytometry-based analysis of the percentage of infection. D) Virion particles retrieved from infected cell cultures expressing or not IFITM3 at the latest time points were purified and then normalized for protein or genome content. The infectivity of normalized virions produced in infected cell cultures expressing or not IFITM3 was then assessed as described above on HeLaP4 (VSV and HIV-1), Vero (EBOV and MeV) or Huh-7.5.1c2 cells (WNV). The graph present averages and SEM of 3 independent experiments. *, p≤0.05 after a Student t test.

To address this issue, replication competent VSV, HIV-1, EBOV, MeV and WNV viruses were used at low MOI to challenge dox-inducible cell lines stably expressing IFITM3 (according to the scheme presented in Fig 7A) and viral spread through dox-induced or uninduced cultures was measured at the indicated time points post infection (Fig 7B). As expected, induction of IFITM3 reduced the spread of every virus tested, although the magnitude of the reduction varied (Fig 7B). To quantify the ability of IFITM3 to protect cells against incoming viruses, cells were challenged at day 0, at a time when secondary infection remains negligible. In this case, high viral doses and a short time analysis of infected cells post challenge were used to appreciate in as much as possible the antiviral effects on a single cycle basis (Fig 7C). Under these conditions and as expected from data in the field, expression of IFITM3 was associated to a statistically significant protection of target cells (from 1.3 to 4.3 fold). Very variable inhibition rates have been reported for different viruses across different studies, in some instances higher than what we describe here. We believe these differences are mainly due to the heterogeneity of the experimental systems used (cell types, IFITM expression levels, use of pseudovirions *versus* replication-competent viruses, time of analysis, etc).

Finally, the effects played by IFITM3 in the production of infectious virions was assessed by harvesting cell free supernatants at late time points during spreading infections. Virion particles were then purified, normalized and their infectivity was assessed on target cells (as in Fig 4) again using high viral doses and short time of analysis after challenge (Fig 7D). Under these conditions, virion particles produced during spreading infection in cell cultures expressing IFITM3 displayed a reduced infectivity when compared to viruses produced in its absence (from 2 to 6 fold), thus confirming in a different experimental system the results presented in Fig 4.

For the first time, the results presented here indicate that the antiviral effects played by IFITM3 during viral replication result from its ability to interfere with two very distinct moments of the viral life cycle and this for viruses as diverse as EBOV and HIV-1.

### The specificity of the virion mode of assembly and not the viral glycoprotein is a determinant factor in the virus susceptibility to IFITMs

In the case of HIV-1, several studies implicate the Envelope glycoprotein in the modulation of the viral susceptibility to IFITMs, in particular through their ability to engage distinct co-receptors during cell entry [23, 47, 48].

**Fig 8:**
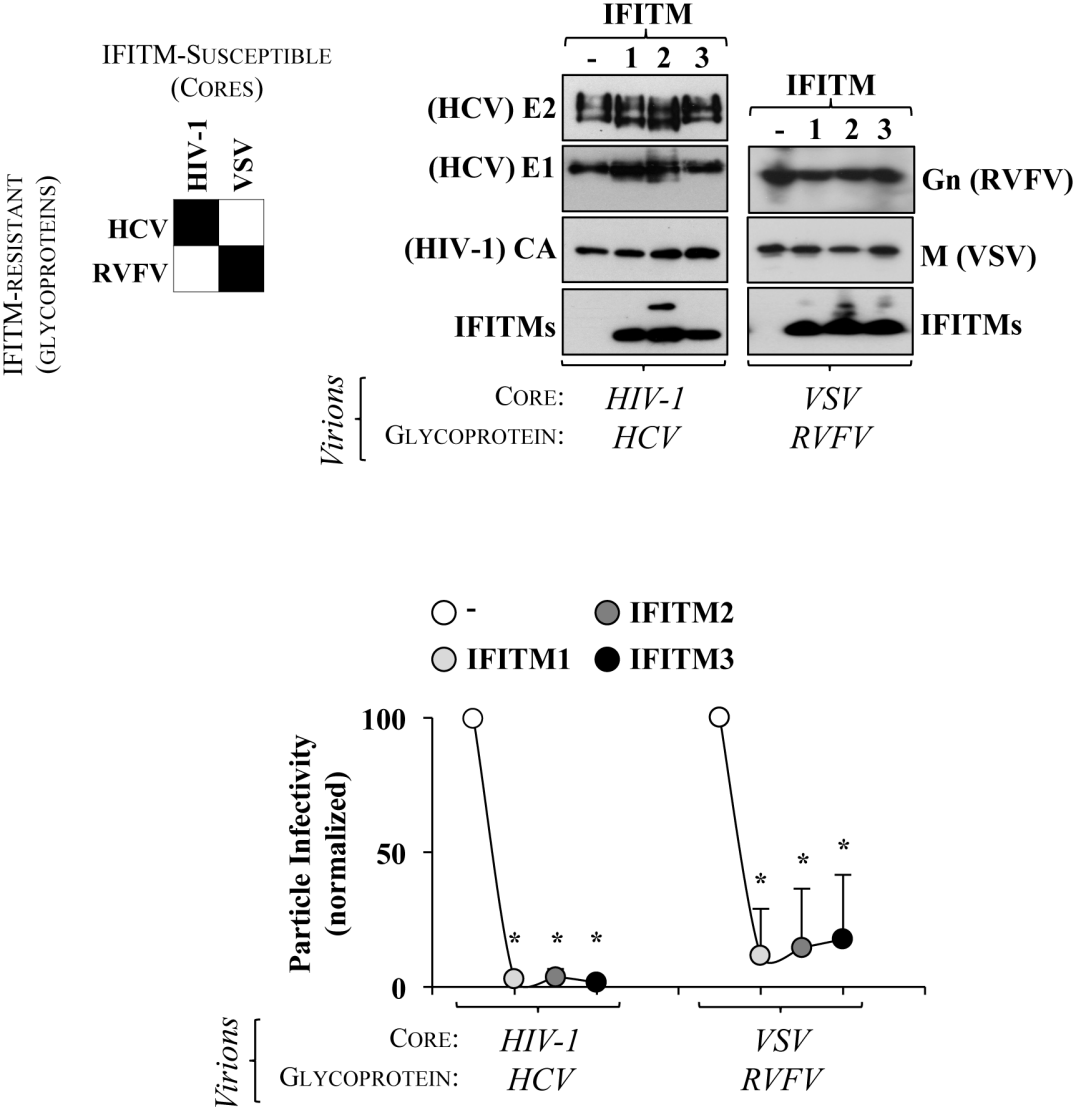
Genetic elements swapping indicates the mode of virion assembly as a dominant determinant in the virus susceptibility to IFITMs. Glycoproteins of IFITM-resistant viruses were used to pseudotype viral cores of IFITM-susceptible viruses (as indicated). HIV-1 virions presenting the HCV E1/E2 glycoproteins were produced in the presence of IFITMs by co-transfection of the respective DNAs (1:1 ratio). VSV pseudoparticles (VSVpp) incorporating the RVFV GnGc in the presence of IFITMs were produced in HEK293T transfected with DNAs coding for GnGc along with IFITMs (0.7:1 ratio), following challenge with a viral stock of ΔG-VSV virus that had been previously complemented with the G protein to allow its entry into cells. After entry, absence of G allows the production of novel virion particles that assembled in the presence of IFITMs and of the RVFV GnGc glycoprotein. Residual input virus was neutralized by incubation with an anti-G neutralizing antibody. Virion particles were purified from the supernatant of virus-producing cells, normalized and used to challenge target cells in a classical single round of infection prior to flow cytometry analysis. The panels present typical results, while the graphs present averages and SEM of 3 to 5 different experiments. *, p≤0.05 after a Student t test.

To determine whether the virion assembly mode or the identity of the glycoprotein could be important determinants in the antiviral effect of IFITMs, we tested heterologous combinations between cores and glycoproteins issued from susceptible or resistant viruses and assembled in the presence or absence of IFITMs (Fig 8). To this end, we pseudotyped HIV-1 cores with the glycoproteins E1-E2 of HCV, system that had been developed before and that is widely used in the field to study HCV entry [60]. In addition, we developed for this study VSV-based cores pseudotyped with the GnGc glycoprotein of RVFV.

Cell free virion particles produced in the presence or absence of IFITMs were purified, normalized and then either analyzed by WB, or used for viral challenge prior to flow cytometry analysis (Fig 8). Under these experimental conditions, IFITMs were well incorporated into both HIV-1 and VSV-based cores. When the infectivity of these virion particles was determined, a very important infectivity defect was observed for HIV-1 and VSV cores bearing the envelope glycoproteins of HCV and RVFV.

Overall, these results strongly suggest that viral specificities in the mode of assembly, rather than a specific glycoprotein represent the dominant factor in the susceptibility of a given virus to IFITMs.

### CCR5 co-receptor usage relieves the negative effects specified by IFITM3 on HIV-1

**Fig 9:**
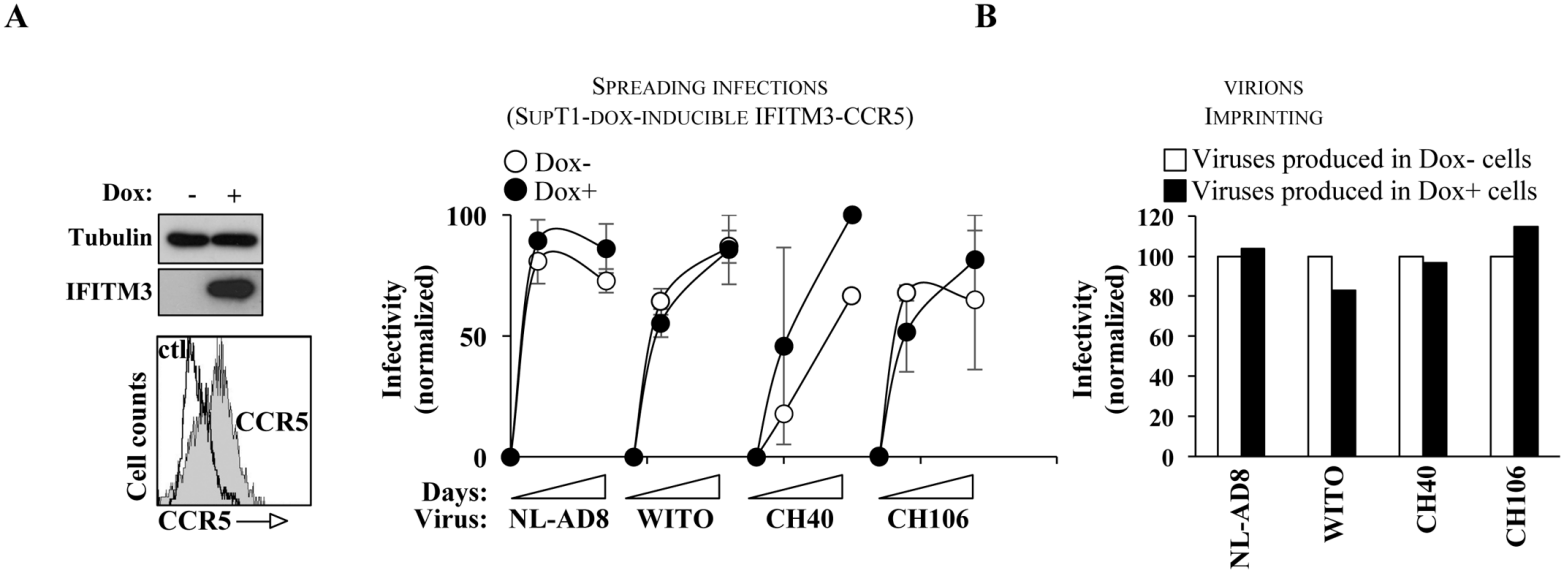
CCR5 usage relieves the negative effects of IFITM3 on HIV-1 replication and on its ability to decrease the virion particles infectivity. A) Human CCR5 was introduced in the IFITM3-stable SupT1 cells used before, by retroviral-mediated gene transduction and cells were challenged with the indicated viruses. HIV spreading was assessed by exo-RT activity over time (day 0 through 7). The panels and the histogram overlay present the patterns of expression obtained for IFITM3 and CCR5 following WB and flow cytometry analyses. The graph presents normalized data obtained in 2 to 3 independent experiments. B) Virions obtained at late times after infection were harvested, normalized and used to infect HeLaP5 cells that contain a β-galactosidase reporter gene under the control of the HIV-1 LTR.

Despite the fact that our genetic swapping results indicated that viral glycoproteins were not dominant determining factors in the susceptibility to IFITMs, several reports have described how HIV-1 envelope proteins may circumvent the negative effects of IFITMs according to their ability to use either CXCR4 or CCR5 as co-receptor molecules [23, 47, 48]. In particular, HIV-1 envelope molecules with R5-tropism have been shown to display near complete resistance to IFITM3 [23, 47, 48]. To determine whether this was the case here, we introduced CCR5 by retroviral-mediated gene transduction in the IFITM3-stable SupT1 cells used in Fig 7 and we challenged these cells with the following R5-tropic HIV-1 strains: NL-AD8 that contains an R5-tropic envelope in the context of an otherwise NL4-3 proviral clone; three transmitted founder viruses that have been obtained by single-genome amplification in [61]. The four viruses tested replicated well irrespectively of the presence of IFITM3 (Fig 9A) and when virion particles produced during spreading infection were isolated, normalized and analyzed for their infectivity on a single cycle of infection basis, no defects were observed (Fig 9B). These results thus, confirm previous reports with respect to the resistance of R5-tropic viruses to IFITM3 [23, 47, 48] and indicate that the ability to use different entry molecules and/or entry pathways may play an important modulatory role in the susceptibility of different viruses towards these restriction factors.

## Discussion

IFITMs have been firmly established as broad antiviral factors that act mainly through the sequestration of infecting virions in endosomes. On the contrary, the existence of an additional mechanism of viral interference with which IFITMs could lead to the formation of virions of reduced infectivity had been previously reported only in the case of HIV-1 [18, 19, 22].

By analyzing a large panel of viruses, we now establish that the latter, that we refer to as the negative imprinting of the virion particles infectivity is a second conserved antiviral property of IFITMs that extends well beyond HIV and that targets different classes of enveloped viruses.

For the first time, we now reveal that this antiviral mechanism concurs with the previously described endosomal retention of particles in target cells to the overall inhibition ascribed to IFITMs during viral replication. Therefore, the results presented in this study contribute to highlight IFITMs as a novel paradigm of restriction factor capable of interfering with both extremes of a virus life cycle. Given that the protection offered by antiviral factors is often incomplete, this dual mechanism of inhibition is likely to potentiate the leverage of IFITMs on viral replication.

**Fig 10:**
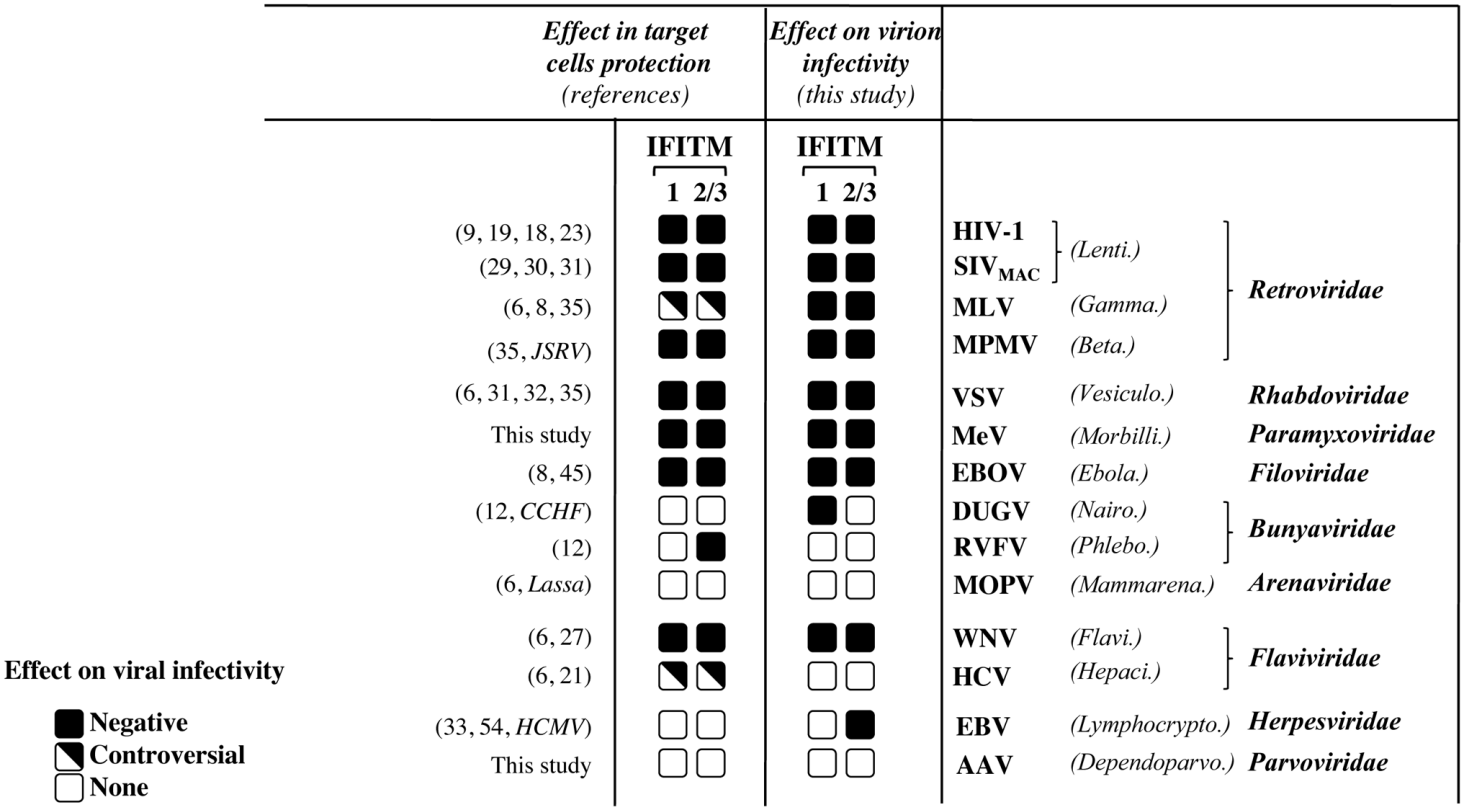
Comparison between the antiviral effect of IFITMs reported in the literature for the different viruses and mediated by the pool of IFITM proteins in target cells, with the negative imprinting of the virion particles infectivity reported in this study. Given their high identity, the antiviral effects of IFITM2 and 3 are presented together, separately from those of IFITM1. Variations in the magnitude of the antiviral effects reported in the different studies have not been taken into account here, as they are likely influenced by the specific experimental conditions used, so that the effects of IFITMs on viral infectivity are presented as negative, absent (none), or controversial, even when a single conflicting report exists. When data in the literature was not directly comparable to ours (i.e. the same virus was not used), data was compared to its closest relative, marked in *italicus*. The effects of the expression of IFITMs in target cells against AAV and MeV were measured in this study and are presented in Supplementary S6 Fig and Fig 7C, respectively.

Despite what seems to be a large spectrum of viral inhibition, our analysis also highlighted the existence of viruses resistant to the three IFITM members tested (HCV, RVFV, MOPV and AAV), as well as of viruses resistant to a given member, but susceptible to others (EBV, DUGV), indicating the existence of a certain degree of heterogeneity in the manner with which viruses cope with IFITMs. This heterogeneity is not surprising, as a large spectrum of behaviors had been previously described in the literature with respect to the antiviral effects played by IFITMs in target cells [6–15, 16, 17, 18, 19, 20, 21, 22, 54, 62]. It is worth noticing that the comparison between the effects reported here and those described in the literature (Fig 10) reveals a largely concordant behavior, albeit with few exceptions likely driven by the heterogeneity of the experimental systems used across studies, in that viruses susceptible to IFITMs in target cells are also so during viral particle production. We believe that this concordance lends support to the previously unappreciated notion that dual inhibition is indeed the general mechanism of inhibition orchestrated by IFITMs.

In our initial description in HIV-1, we had determined that IFITMs were incorporated into virion particles that displayed decreased infectivity [18]. It remains however unclear whether the physical incorporation of IFITMs into virion particles is required for their antiviral effect, or whether these two properties can be dissociated. While a thorough mutagenic study of IFITM proteins that may dissociate the two properties will likely be required to formally address this question, several lines of evidence stemming from our comparative study support the notion that the incorporation of IFITMs into virions can indeed be separated from their antiviral effects. First, virion packaging is not predictive of the antiviral effects of IFITMs (i.e. it is not sufficient), as HCV, RVFV, MOPV, as well as EBV and DUGV in the case of specific IFITM members, do not display detectable infectivity defects. Second, an antiviral effect can be observed even in the absence of IFITM incorporation, as is the case for DUGV virions produced in the presence of IFITM1. These considerations lend support to a model whereby IFITM-packaging and IFITM-mediated imprinting of viral particles can be at least in some cases uncoupled and suggests that the imprinting effect of IFITMs takes place in the cell itself, during the process of virion particles formation.

IFITMs are membrane-associated proteins and therefore their presence in exosomes, in addition to virion particles is not overall surprising. Our CD45-depletion experiments indicate however that in cells undergoing virion assembly, the majority of IFITM proteins is virion-associated, supporting our immuno-gold EM results and excluding *de facto*, a confounding role for contaminating exosomes in our infectivity assays.

In our study, we have noted that viruses assembling for example at the plasma membrane are similarly affected by the three IFITMs (albeit changes in the magnitude of the defects were observed in some cases), despite reported differences in the intracellular distributions among IFITM members [1, 6, 16, 20, 36, 37, 63]. While it remains possible that the experimental setup used here may not have allowed us to finely appreciate differences between individual IFITM members, our results indicate that even the most internally distributed IFITM members (IFITM2 and 3) are clearly present at the plasma membrane of non-permeabilized cells. This suggests that the steady state distribution of IFITM proteins may provide only a limited overview of what is likely to be instead a more complicated process. Indeed, virion assembly and membrane trafficking are highly dynamic processes and the very association of IFITMs to membranes is regulated by an intricate network of regulatory modifications and interactions with cellular co-factors (adaptor protein 2, AP2, the E3-ubiqutin ligase neural precursor cell expressed developmentally down-regulated protein 4, NEDD4, Fyn [20, 36, 37]. In light of these considerations, we do believe that the complex dynamics of membrane trafficking are likely to facilitate the encounter between individual IFITM members and viruses assembling at different intracellular locations, likely explaining the results obtained here.

The heterogeneous behavior of different viruses towards IFITMs allowed us to provide mechanistic insights into the viral determinants of susceptibility to this family of restriction factors through the swapping of genetic elements between IFITM-resistant and susceptible viruses. Despite our intensive efforts not all viral systems proved amenable to functional swapping, so that we used a previously described pseudo-particle system based on an HIV core incorporating E1/E2-HCV glycoproteins [60] and we developed a novel one based on a VSV core packaging GnGc-RVFV. The results we have obtained indicate that the mode of virion assembly intended here as the plethora of viral proteins that orchestrate the production of novel virion particles constitute the dominant determinant of susceptibility to IFITMs. In this respect, the possibility that IFITM-resistant viruses may code for natural antagonists of IFITMs is of high potential interest. The data obtained in our study seem to exclude a degradation-based antagonism, since no detectable depletion of IFITMs is observed in either cell- or virion-associated samples of HCV, RVFV or MOPV viruses. However, antagonism can be established in other manners for instance through the induction of specific post-translational modifications that affect the normal antiviral behavior of IFITMs. At present, this hypothesis remains of interest, but requires further experimental work.

Despite the fact that our results exclude the viral glycoprotein, and modifications thereof, as dominant factors in the viral behavior towards IFITMs, recent studies have described how the HIV-1 Envelope tropism, i.e. its ability to engage the CXCR4 or the CCR5 co-receptors, can modulate the susceptibility to IFITMs, in some cases relieving inhibition completely [23, 47, 64]. The results obtained here with R5-tropic viruses also concur with this conclusion, indicating that R5-tropic viruses resist IFITM3, at least when expressed in T cells [23, 47, 64]. At present, it remains unclear how entry through CCR5 rather than CXCR4 would be so different as to drive such opposite outcomes, but two further considerations may help completing the canvas. First, co-receptor usage is often coupled with different affinities for CD4, so that both parameters may be of importance with respect to the behaviour towards IFITMs. Second, in our previous study we have shown that endogenous IFITMs silencing in primary macrophages increases the infectivity of R5-tropic HIV-1 [23, 47, 64], indicating that co-receptor usage and infection outcome may also be finely dependent on the cell type. This would not be unprecedented as for instance primary macrophages resist X4-tropic virus infection despite the presence of the appropriate co-receptor. Taken together, we believe that these results indicate that changes in co-receptor usage coupled to different affinities for CD4 may influence the outcome of infection with respect to IFITMs in a cell type dependent manner. Whether this occurs or not remains to be determined, however these experimental observations certainly reveal the presence of an additional layer of complexity in the relationship between HIV and IFITMs that it would be of interest to explore in other viral settings.

While we have not measured it directly due to the lack of robust viral entry systems for most of the viruses used here, IFITMs have always been reported to act by altering the fusogenic properties of cellular or of viral membranes with their opposite counterpart [18, 19, 22, 34, 35, 43]. Considering that viral membranes are ultimately derived from the cell, we favor the hypothesis according to which IFITMs act using a single molecular mechanism that modifies cellular membranes to yield to two distinct outcomes: an effect in target cells and an effect on newly-produced virions. Not much is known about the molecular mechanisms through which IFITMs act, but at least two interesting cellular proteins have been described to be potentially involved in their antiviral effects: VAPA, a key regulator of the lipid composition of cellular membranes that is posited at ER-Golgi interfaces [44] and more recently ZMPSTE24 that acts as an endoprotease within the cell [46]. The role of these cellular co-factors in the antiviral mechanisms of inhibition of IFITMs remains to be wholly elucidated, but it is easy to envision how modifications in either the protein or lipid composition of cellular (and therefore viral) membranes could provide a broad mechanism of interference with a large spectrum of viruses. If this hypothesis is correct, the protection provided by some HIV-1 glycoproteins Env may be due to their increased propensity to acquire a fusion-prone conformation or to their lower requirement for intramembrane movements upon the engagement of the appropriate cellular receptors.

In conclusion, this study indicates that the ability of IFITMs to interfere with the production of infectious viral particles is a conserved antiviral property of IFITMs and highlights for the first time IFITMs as restriction factors capable of interfering with viral replication at two distinct moments of their life cycle.

## Material and methods

### Cells

HEK293T, HeLa, A549, SupT1 and Vero cells, obtained from the Cellulonet repository of the SFR-Biosciences Gerland, were maintained in complete DMEM media supplemented with 10% FCS (SIGMA; RPMI 1640 media for SupT1). Huh-7.5.1c2 cells were similarly propagated and were obtained from the laboratory of Francis V. Chisari (the Scripps Research Institute, La Jolla, CA, USA [65]. Hone cells that contain a latent EBV-GFP genome were maintained in RPMI 1640 [50]. Vero/hSLAM cells expressing the human CD150 receptor (SLAM) were used for most Measles Virus infections/productions [66] and were obtained from Yusuke Yanagi (Kyushu University, Fukuoka, Japan). HeLaP4 cells stably expressing the HIV-1 CD4 receptor and CXCR4 co-receptor in addition to an integrated *ß-galactosidase* under the control of the HIV-1 Long Terminal Repeats promoter have been described before [67].

Peripheral blood lymphocytes (PBLs) were obtained after Ficoll gradient purification. Cells were maintained in RPMI 1640 supplemented with 10% FCS. Prior to usage, PBLs were activated for twenty-four hours with 1 μg/ml of phytohemagglutinin (PHA, Sigma) and 150 U/mL of Interleukin 2 (IL2, obtained through the AIDS Reagents and Reference Program of the NIH). Monocytes were first enriched from white blood leukocytes through successive Ficoll and Percoll gradients and then purified by negative depletion (monocyte isolation kit II, catalogue n° 130-091-153, Miltenyi) to obtain a cell population of purity equal/superior to 95%. Monocytes were either differentiated in immature monocyte-derived dendritic cells (MDDCs) after incubation for 4 days with GM-CSF and IL4 (each at 100 ng/mL each, catalogues n° PCYT-221 and PCYT-211, Eurobio) or in macrophages upon incubation with 100 ng/mL of M-CSF (Eurobio catalogues n° 01-A0220-0050). When indicated, MDDCs were incubated for 24 hours with 1.000 U/mL of human IFNα (catalogue n° 11100–1, Tebu Bio).

### Ethics statement

Primary blood cells were obtained from the blood of healthy donors (EFS-Lyon) in the form of discarded “leukopacks” obtained anonymously so that gender, race, and age of donors are unknown to the investigator and inclusion of women, minorities or children cannot be determined. This research is exempt from approval, although written informed consent was obtained from blood donors to allow use of their cells for research purposes.

### Plasmids, viral systems and antibodies

A complete overview of all the viruses and viral systems used here is provided in Supplementary S1 Fig and S1 and S2 Tables. Antibodies were either acquired, or provided as described in the Supplementary S2 Table and in the following references [68–71].

N-terminal Flag-tagged IFITM1, 2 and 3 coding DNAs have been described in [18] and untagged version of these proteins were obtained by standard cloning techniques. To circumvent the poor transfection rates of Huh-7.5.1c2 cells, retroviral transduction was used to generate cells that expressed Flag-tagged IFITMs in a doxycycline-inducible form as in [9, 18]. The EBV-EB1 as well as the HCV E1/E2 (genotype 1, H77) expressing plasmids have been described in [49] and [72], respectively. The RVFV GnGc coding sequence was cloned in the context of a pHCMV construct using standard molecular biology techniques.

Retroviral vectors coding the human CCR5 were obtained through the AIDS repository of the NIH. When indicated, CCR5-stably expressing cells were obtained by retroviral mediated gene transduction. The anti-CCR5 antibody used for flow cytometry was purchased from Becton Dickinson.

### Viral production

A schematic overview is presented in Supplementary S1 Fig.

- Retroviral particles derived from HIV-1, SIV_MAC_, MLV and MPMV [73–76] were produced in the presence or absence of IFITMs by calcium phosphate DNA co-transfection of HEK293T cells. Retroviral particles are produced using the following ratios, as described: 12:4:4:2 of DNAs coding for IFITMs, the structural polyproteins Gag-Pro-Pol, a miniviral genome bearing a GFP reporter and the envelope protein G of the Vesicular Stomatitis virus (VSV-G) that confer broad viral tropism to the retroviral particles thus produced. In the case of MPMV, the ratio was instead 12:8:2, given that MPMV-derived vectors are based on two and not three plasmids: a viral genome bearing *gfp* in the place of *env* and envelope provided separately.
- AAV-derived viral particles [77] were also produced by co-transfection of DNAs coding for IFITMs as well as for the AAV Rep proteins and cognate *gfp*-bearing genome at a ratio of 12:8:4, respectively. In this case to ascertain the true absence of IFITMs in viral preparations, virions were produced from four times more starting material than used for retroviruses.
- WNV virions were produced by co-transfection of DNAs coding a cloned WNV replicative genome bearing *gfp* [78] along with IFITMs (10 and 12 μg of DNA for a 10 cm plate, respectively).
- For VSV [79], MeV [80], EBOV [81], HCV [82], DUGV [83], RVFV [84] and MOPV [85], for which replicative viruses were available, the following procedure was used. Target cells (indicated in Supplementary S1 Fig) were first transfected with DNA coding for IFITMs, then challenged with replicative virus four to six hours post transfection. The short interval of time between IFITMs-coding DNA transfection and viral challenge ensures the unrestrained entry of these viruses in target cells due to the low to undetectable levels of IFITMs at this early time point. Upon extensive cell washing to remove the input virus, this setup allows the establishment of a large proportion of IFITMs-expressing, virus-producing cells.
- EBV virions were produced by transfecting Hone cells that bear a latent EBV genome coding GFP [50] with DNAs coding for IFITMs along with the viral immediate early transcription factor EB1. Under these conditions, EB1 re-activates the lytic cycle of EBV and the production of novel viral particles.

In all cases, virions produced in the presence/absence of IFITMs were collected 48 to 72 hours after the initial viral challenge or transient transfection.

When indicated, replicative experiments were performed with NL-AD8, an HIV-1 construct in which the NL4-3 envelope has been replaced for the corresponding one of the R5 tropic HIV-1 strain ADA [86], and with HIV-1 transmitted founder strains WITO, CH40 and CH77 (Supplementary S1 Table, [61]). Infectivity of virion particles was measured in this case in HeLaP5, a reporter cell line that bears the CCR5 co-receptor and the β-galactosidase gene under the control of the viral LTR.

HIV-1 virion particles pseudotyped with the HCV E1/E2 glycoproteins have been described before [72]. For the preparation of VSV pseudotypes, HEK293T cells were first transfected with DNAs expressing RVFV-GnGc and IFITMs. Then, twenty-four hours later cells were infected with a viral stock of replication-deficient ΔG-VSV virus previously complemented with VSV-G [87]. VSV-G reconstitution allows viral entry into the cell and expression of the VSV genome with the exception of the G gene, to allowing the production of pseudoparticles incorporating the glycoprotein of interest. One hour after infection, residual viruses were neutralized with 1hr incubation with the monoclonal antibody (mb4IAI) directed against VSV-G. One day later, the cell supernatant was collected and clarified by centrifugation at 600*g*.

### Virus purification

With the exception of AAV (see below), cell free supernatants were first centrifuged at low speed (5 minutes, 1,000g) and syringe-filtered (0.45 μm). Then virion particles were purified by ultracentrifugation through a 25% sucrose cushion (w/vol) for two hours at 110.000g. Viral pellets were then resuspended in PBS for further analysis.

Since AAV virions assemble inside the nucleus, cells were lysed in 150 mM NaCl, 50 mM Tris/HCl, pH 8.5 and subjected to 3 rounds of freeze/thaw (liquid nitrogen -37°C, with robust vortexing after each thawing step). The crude lysate was clarified by centrifugation at 1.000g, then at 10.000g (5 minutes each). The supernatant was then purified by ultracentrifugation at 63.000g for 1 hour on a iodixanol four-step gradient (15%, 25%, 40%, 60%). After centrifugation AAV was retrieved in the 40% iodixanol fraction, as described [51].

### CD45-depletion

SupT1 cells stably transduced with individual dox-inducible IFITM members were infected with HIV-1 (2x10^6^ cells, MOI 0.5; 1 week in culture) or with VSV (2x10^6^ cells, MOI 0.05; 2 days in culture). At the end of the culture period, virions were harvested, syringe-filtered and divided in two fractions that were either left untreated or incubated with 50 μL of CD45-conjugated microbeads (Miltenyi, catalogue# 130-045-801) for 1 hour at 4°C in constant nutation. Microbeads were then passed through a retaining column (Miltenyi, catalogue#130-042-201) and the flow through was harvested. Virion particles contained in both CD45-depleted and non-depleted fractions were then purified by ultracentrifugation through sucrose, normalized by exo-RT activity and compared by WB and infectivity analyses. The anti-CD45 antibody used in WB was purchased by Becton Dickinson (catalogue# 610266).

### Lentiviral vector-mediated miR30-shRNAs silencing of IFITMs

Silencing of endogenous IFITMs was obtained by miR30-shRNAs after the stable transduction of recipient cells with an HIV-1-based lentiviral vector as described in [18]. Lentiviral vectors were produced by cotransfection of HEK293T cells with DNAs coding the structural proteins Gag-Pol and Envelope plus a viral genome bearing a miR30-shRNAs-Puromycin cassette. Two target sequences per IFITM were used, for a total of six co-transfected in the same HEK293T cell plate to obtain a single viral preparation for either control or IFITM silencing. Virion particles were then purified as described above and normalized by exogenous-reverse transcription assay. Normalized amount of lentiviral vectors were used on target cells to obtain silencing. In the case of HeLa cells and activated PBLs, cells were challenged with a multiplicity of infection comprised between 1 and 2 (MOI, the infectious titers of the viral vectors were established upon comparison with standards of known infectivity) for three rounds of viral challenge (every twenty-four hours). Cells were then briefly selected with Puromycin for two days to enrich the cell culture in effectively silenced cells.

In the case of macrophages, cells were challenged only once with an MOI of 1 of miR30-shRNAs coding viruses in the presence of an MOI-equivalent of 0.5 of virion-like particles bearing the SIV_MAC_ Vpx (or VLPs-Vpx). This trick allows a very efficient transduction of myeloid cells by HIV-1 [88–90] thanks to the reported ability of Vpx to remove the restriction factor SAMHD1 [91, 92]. The target sequences used were published before by our laboratory [18] and are reported here uniquely for ease: luciferase (acc.n° DQ188838): AGCTCCCGTGAATTGGAATCC; IFITM1 (acc.n° NM_003641.3): ATCTGTGACAGTCTACCATATT and CCCATATTATGTTACAGATAAT; IFITM2 (acc.n° NM_006435.2): ACCAGCCTCCCAACTACGAGAT and ACCCGATGTCCACCGTGATCCA; IFITM3 (acc.n° NM_021034.2): ACCCGACGTCCACCGTGATCCA and ACCCCCAACTATGAGATGCTCA.

### Normalization of viral particles

Virion particles produced in the presence or absence of the different IFITMs were normalized as described in Supplementary S1 Fig and S2 Table.

- HIV, SIV_MAC_ and MLV particles were normalized by exogenous-reverse transcriptase, a quantitative assay that measures the amount of viral-associated RT enzyme through its ability to incorporate radioactive ^32^P TTP in a poly rA matrix/oligo dT template [90]. This assay did not work on MPMV, so that MPMV particles were normalized after SDS-PAGE and Coomassie quantification of the viral CA protein, as no specific antibodies were available for this study.

The remaining viruses were normalized by qPCR or RT-qPCR using genome specific primers, as listed in S2 Table.

### Measurement of viral particles infectivity and spreading assays

Infections were carried out on normalized viral preparations using the different virus-specific assays, as detailed in Supplementary S1 Fig. A comparative overview of the infectious viral titers obtained and used is presented in Supplementary S3 Fig. Infectivity of single round competent HIV-1, SIV_MAC_, MLV, MPMV, EBV and AAV viral particles was measured on HEK293T cells, or on B lymphocytic Raji cells for EBV, by FACS three days after infection. To approximate a single cycle of infection, infections with the replication competent WNV, VSV, MeV and RVFV viruses were carried out at high MOI and the percentage of infected cells was measured at the shortest detectable time point by FACS to avoid multiple cycles of infection (16, 8, 24 and 6 hours post infection, respectively for MOI comprised between 0.1 and 0.5). In all cases accumulation of the viral coded GFP was measured in target cells with the exception of RVFV for which NSs was measured.

Spreading infections were carried out at low MOI (for VSV, HIV-1, EBOV, MeV and WNV from 0.01 to 0.2 depending on the virus) and the progression of the infection was monitored through the accumulation of GFP-positive cells over time by FACS.

Focus forming assays (FFA) or 50% tissue culture infectious dose (TCID50) assays were used in the case of HCV, DUGV, MOPV and in some cases EBOV, respectively.

### Immuno-gold electron microscopy

Formvar/carbon-coated nickel grids were deposited on a drop of purified, unfixed viruses produced in the presence or absence of Flag-tagged IFITM3 for five minutes prior to sequential incubation with an anti-Flag antibody (F7425, Sigma, St-Louis, MO), followed by incubation with a 1:30 gold-conjugated (10 nm) goat-anti-Rabbit IgG (Aurion, Wageningen, Netherlands) and fixation in 1% glutaraldehyde. Negative staining was performed using 2% uranyl acetate (Agar Scientific, Stansted, UK) followed by, transmission electron microscope analyses (JEOL 1011, Tokyo, Japan).

## Supporting information

S1 Table Viruses and viral systems used in this study. (DOCX)

S2 Table List of antibodies and oligonucleotides used in this study. (PDF)

S1 Fig Overview of the specific experimental setup used. The figure presents schematically the major information on the assays used for each virus to generate the data presented in this study.
(TIF)

S2 Fig Quantification of the amount of Flag-tagged IFITMs in virus-producing cells and comparison with endogenous IFITMs. A) To determine the level of Flag-tagged IFITM proteins present upon transient transfection in the different virus-producing cells, cells were analyzed by intracellular staining with an anti-Flag antibody, followed by flow cytometry. Representative panels are shown on top, while quantification of the number of positive cells and their median fluorescence intensity (MFI, that relates to the intracellular levels of IFITM proteins present in positive cells) are shown as graphs. B) The intracellular levels of IFITMs expressed upon transient transfection of the highest expressing cells (HEK293T-transfected with Flag-IFITM3) were compared to the endogenous levels present in monocyte-derived dendritic cells (MDDCs) stimulated for 24 hours with 1.000 U/ml of IFNα, using antibodies directed against IFITM2 and IFITM3. The top panels present typical results obtained in three different donors, while the graph at the bottom compares the MFI obtained (for 6 different donors). Endogenous as well as ectopically-expressed IFITM2/3 proteins are detectably present at the cell surface. C) Monocyte-derived dendritic cells (MDDCs) stimulated for twenty-four hours with IFNα were analyzed by flow cytometry with anti-IFITM2 and IFITM3 antibodies with or without prior cellular permeabilization. IFITM1 is not shown here, because the anti-IFITM1 antibodies we examined did not yield reliable staining in flow cytometry. D) Membrane staining profiles of Flag-tagged IFITM1, 2 and 3 ectopically expressed in the different cell types used in this study. The smaller shift detected in Hone cells is likely due to the lower transfection rate and intracellular accumulation level of IFITMs in this cell line, rather than to a cell type-specific behavior.
(TIF)

S3 Fig A and B) Comparison of the infectious titers used in this study for the different viruses, as estimated by flow cytometry or FFA/TCID50. The two graphs compare the average infectivity of viral preparations used throughout this study as determined by FACS (in the case of most *gfp*-bearing viruses), or by FFA/TCID50 (presented here as infectious units/ml). C) Effects of IFITMs on the production of viral particles. The viruses produced in the presence or absence of IFITMs were quantified according to the methods presented in the Supplementary S1 Fig. For each virus, values have been normalized to control virions produced in the absence of IFITMs. The graph presents the averages of 3 to 4 distinct experiments.
(TIF)

S4 Fig A) Comparison of the antiviral effects exerted by tagged and natural IFITM proteins. HIV-1 and VSV viral particles were produced in cells transfected with DNAs coding Flag-tagged and non-tagged IFITMs. The panels display typical expression patterns of virus-producing cells, while the graph reports averages and SEM obtained from 4 independent experiments. No statistically significant difference was observed between the effects of individual pairs of IFITMs following a Student t test. B) Determination of the antiviral effects of IFITM1 in HuH7 against VSV. To determine whether the lack of antiviral effects observed for HCV virions produced in HuH7 cells in the presence of the different IFITMs presented in Fig 4 was not due to insufficient expression of IFITMs, HuH7 cells expressing IFITM1 (the least expressed among the IFITM members) were used as VSV-producing cells as indicated in the scheme above. Newly-produced viral particles were then normalized and used to challenge HEK293T cells. The graph presents averages and SEM of 3 independent experiments. * = p≤0.05, after a Student t test.
(TIF)

S5 Fig Replication of EBOV in primary monocyte-derived macrophages silenced for IFITMs. This figure presents the complete analysis of EBOV replication in primary monocyte-derived macrophages (due to space constraints only GFP-positive cells obtained at day 2 are presented in Fig 5). Briefly, blood monocytes were differentiated in macrophages upon incubation with M-CSF during four days in a 24 well-plate format. Cells were then transduced with an MOI of 1 of HIV-1 vectors bearing an shRNA expression cassette directed against control sequences (Luciferase) or against IFITM1, 2 and 3. Four days afterwards, cells were challenged with an MOI of 0.3 of EBOV. Viral spread was analyzed through the accumulation of GFP-positive cells thanks to the virus-coded GFP reporter.
(TIF)

S6 Fig Effect of IFITM proteins expressed in target cells on incoming AAV viral particles. Cells expressing IFITM1, 2 and 3 were challenged with GFP-coding AAV, prior to the quantification of the percentage of GFP-positive cells by flow cytometry. The graph present averages and SEM of 3 independent experiments.
(TIF)

## References

[1] Bailey CC, Kondur HR, Huang IC, Farzan M. Interferon-induced transmembrane protein 3 is a type II transmembrane protein. J Biol Chem. 2013;288(45):32184–93. Epub 2013/09/27. doi: 10.1074/jbc.M113.514356 . 24067232 10.1074/jbc.M113.514356 PMC3820858

[2] Weston S, Czieso S, White IJ, Smith SE, Kellam P, Marsh M. A membrane topology model for human interferon inducible transmembrane protein 1. PLoS One. 2014;9(8):e104341 Epub 2014/08/12. doi: 10.1371/journal.pone.0104341 . 25105503 10.1371/journal.pone.0104341 PMC4126714

[3] Friedman RL, Manly SP, McMahon M, Kerr IM, Stark GR. Transcriptional and posttranscriptional regulation of interferon-induced gene expression in human cells. Cell. 1984;38(3):745–55. . 6548414 10.1016/0092-8674(84)90270-8

[4] Farber CR, Reich A, Barnes AM, Becerra P, Rauch F, Cabral WA, et al A Novel IFITM5 Mutation in Severe Atypical Osteogenesis Imperfecta Type VI Impairs Osteoblast Production of Pigment Epithelium-Derived Factor. J Bone Miner Res. 2014 Epub 2014/02/13. doi: 10.1002/jbmr.2173 . 24519609 10.1002/jbmr.2173 PMC4352343

[5] Kasaai B, Gaumond MH, Moffatt P. Regulation of the bone-restricted IFITM-like (Bril) gene transcription by Sp and Gli family members and CpG methylation. J Biol Chem. 2013;288(19):13278–94. Epub 2013/03/27. doi: 10.1074/jbc.M113.457010 . 23530031 10.1074/jbc.M113.457010 PMC3650367

[6] Brass AL, Huang IC, Benita Y, John SP, Krishnan MN, Feeley EM, et al The IFITM proteins mediate cellular resistance to influenza A H1N1 virus, West Nile virus, and dengue virus. Cell. 2009;139(7):1243–54. Epub 2010/01/13. doi: 10.1016/j.cell.2009.12.017 . 20064371 10.1016/j.cell.2009.12.017 PMC2824905

[7] Everitt AR, Clare S, Pertel T, John SP, Wash RS, Smith SE, et al IFITM3 restricts the morbidity and mortality associated with influenza. Nature. 2012;484(7395):519–23. Epub 2012/03/27. doi: 10.1038/nature10921 . 22446628 10.1038/nature10921 PMC3648786

[8] Huang IC, Bailey CC, Weyer JL, Radoshitzky SR, Becker MM, Chiang JJ, et al Distinct patterns of IFITM-mediated restriction of filoviruses, SARS coronavirus, and influenza A virus. PLoS Pathog. 2011;7(1):e1001258 Epub 2011/01/22. doi: 10.1371/journal.ppat.1001258 . 21253575 10.1371/journal.ppat.1001258 PMC3017121

[9] Lu J, Pan Q, Rong L, He W, Liu SL, Liang C. The IFITM proteins inhibit HIV-1 infection. J Virol. 2011;85(5):2126–37. Epub 2010/12/24. doi: 10.1128/JVI.01531-10 . 21177806 10.1128/JVI.01531-10 PMC3067758

[10] Schoggins JW, Wilson SJ, Panis M, Murphy MY, Jones CT, Bieniasz P, et al A diverse range of gene products are effectors of the type I interferon antiviral response. Nature. 2011;472(7344):481–5. Epub 2011/04/12. doi: 10.1038/nature09907 . 21478870 10.1038/nature09907 PMC3409588

[11] Perreira JM, Chin CR, Feeley EM, Brass AL. IFITMs restrict the replication of multiple pathogenic viruses. J Mol Biol. 2013;425(24):4937–55. Epub 2013/10/01. doi: 10.1016/j.jmb.2013.09.024 . 24076421 10.1016/j.jmb.2013.09.024 PMC4121887

[12] Mudhasani R, Tran JP, Retterer C, Radoshitzky SR, Kota KP, Altamura LA, et al IFITM-2 and IFITM-3 but not IFITM-1 restrict Rift Valley fever virus. J Virol. 2013;87(15):8451–64. Epub 2013/05/31. doi: 10.1128/JVI.03382-12 . 23720721 10.1128/JVI.03382-12 PMC3719792

[13] Everitt AR, Clare S, McDonald JU, Kane L, Harcourt K, Ahras M, et al Defining the range of pathogens susceptible to Ifitm3 restriction using a knockout mouse model. PLoS One. 2013;8(11):e80723 Epub 2013/11/28. doi: 10.1371/journal.pone.0080723 . 24278312 10.1371/journal.pone.0080723 PMC3836756

[14] Anafu AA, Bowen CH, Chin CR, Brass AL, Holm GH. Interferon-inducible transmembrane protein 3 (IFITM3) restricts reovirus cell entry. J Biol Chem. 2013;288(24):17261–71. Epub 2013/05/08. doi: 10.1074/jbc.M112.438515 . 23649619 10.1074/jbc.M112.438515 PMC3682530

[15] Bailey CC, Huang IC, Kam C, Farzan M. Ifitm3 limits the severity of acute influenza in mice. PLoS Pathog. 2012;8(9):e1002909 Epub 2012/09/13. doi: 10.1371/journal.ppat.1002909 . 22969429 10.1371/journal.ppat.1002909 PMC3435252

[16] John SP, Chin CR, Perreira JM, Feeley EM, Aker AM, Savidis G, et al The CD225 domain of IFITM3 is required for both IFITM protein association and inhibition of influenza A virus and dengue virus replication. J Virol. 2013;87(14):7837–52. Epub 2013/05/10. doi: 10.1128/JVI.00481-13 . 23658454 10.1128/JVI.00481-13 PMC3700195

[17] Zhang W, Zhang L, Zan Y, Du N, Yang Y, Tien P. Human respiratory syncytial virus infection is inhibited by IFN-induced transmembrane proteins. J Gen Virol. 2015;96(Pt 1):170–82. doi: 10.1099/vir.0.066555-0 . 25228491 10.1099/vir.0.066555-0

[18] Tartour K, Appourchaux R, Gaillard J, Nguyen XN, Durand S, Turpin J, et al IFITM proteins are incorporated onto HIV-1 virion particles and negatively imprint their infectivity. Retrovirology. 2014;11:103 doi: 10.1186/s12977-014-0103-y . 25422070 10.1186/s12977-014-0103-y PMC4251951

[19] Compton AA, Bruel T, Porrot F, Mallet A, Sachse M, Euvrard M, et al IFITM proteins incorporated into HIV-1 virions impair viral fusion and spread. Cell Host Microbe. 2014;16(6):736–47. doi: 10.1016/j.chom.2014.11.001 . 25464829 10.1016/j.chom.2014.11.001 PMC7104936

[20] Jia R, Pan Q, Ding S, Rong L, Liu SL, Geng Y, et al The N-terminal region of IFITM3 modulates its antiviral activity by regulating IFITM3 cellular localization. J Virol. 2012;86(24):13697–707. Epub 2012/10/12. doi: 10.1128/JVI.01828-12 . 23055554 10.1128/JVI.01828-12 PMC3503121

[21] Narayana SK, Helbig KJ, McCartney EM, Eyre NS, Bull RA, Eltahla A, et al The Interferon-induced Transmembrane Proteins, IFITM1, IFITM2, and IFITM3 Inhibit Hepatitis C Virus Entry. J Biol Chem. 2015;290(43):25946–59. doi: 10.1074/jbc.M115.657346 . 26354436 10.1074/jbc.M115.657346 PMC4646249

[22] Yu J, Li M, Wilkins J, Ding S, Swartz TH, Esposito AM, et al IFITM Proteins Restrict HIV-1 Infection by Antagonizing the Envelope Glycoprotein. Cell Rep. 2015;13(1):145–56. doi: 10.1016/j.celrep.2015.08.055 . 26387945 10.1016/j.celrep.2015.08.055 PMC4602366

[23] Foster TL, Wilson H, Iyer SS, Coss K, Doores K, Smith S, et al Resistance of Transmitted Founder HIV-1 to IFITM-Mediated Restriction. Cell Host Microbe. 2016;20(4):429–42. doi: 10.1016/j.chom.2016.08.006 . 27640936 10.1016/j.chom.2016.08.006 PMC5075283

[24] Weston S, Czieso S, White IJ, Smith SE, Wash RS, Diaz-Soria C, et al Alphavirus Restriction by IFITM Proteins. Traffic. 2016;17(9):997–1013. doi: 10.1111/tra.12416 . 27219333 10.1111/tra.12416 PMC5025721

[25] Munoz-Moreno R, Cuesta-Geijo MA, Martinez-Romero C, Barrado-Gil L, Galindo I, Garcia-Sastre A, et al Antiviral Role of IFITM Proteins in African Swine Fever Virus Infection. PLoS One. 2016;11(4):e0154366 doi: 10.1371/journal.pone.0154366 . 27116236 10.1371/journal.pone.0154366 PMC4846163

[26] Savidis G, Perreira JM, Portmann JM, Meraner P, Guo Z, Green S, et al The IFITMs Inhibit Zika Virus Replication. Cell Rep. 2016;15(11):2323–30. doi: 10.1016/j.celrep.2016.05.074 . 27268505 10.1016/j.celrep.2016.05.074

[27] Gorman MJ, Poddar S, Farzan M, Diamond MS. The Interferon-Stimulated Gene Ifitm3 Restricts West Nile Virus Infection and Pathogenesis. J Virol. 2016;90(18):8212–25. doi: 10.1128/JVI.00581-16 . 27384652 10.1128/JVI.00581-16 PMC5008082

[28] Poddar S, Hyde JL, Gorman MJ, Farzan M, Diamond MS. The Interferon-Stimulated Gene IFITM3 Restricts Infection and Pathogenesis of Arthritogenic and Encephalitic Alphaviruses. J Virol. 2016;90(19):8780–94. doi: 10.1128/JVI.00655-16 . 27440901 10.1128/JVI.00655-16 PMC5021394

[29] Wilkins J, Zheng YM, Yu J, Liang C, Liu SL. Nonhuman Primate IFITM Proteins Are Potent Inhibitors of HIV and SIV. PLoS One. 2016;11(6):e0156739 doi: 10.1371/journal.pone.0156739 . 27257969 10.1371/journal.pone.0156739 PMC4892622

[30] Qian J, Le Duff Y, Wang Y, Pan Q, Ding S, Zheng YM, et al Primate lentiviruses are differentially inhibited by interferon-induced transmembrane proteins. Virology. 2015;474:10–8. doi: 10.1016/j.virol.2014.10.015 . 25463599 10.1016/j.virol.2014.10.015 PMC4581848

[31] Wrensch F, Hoffmann M, Gartner S, Nehlmeier I, Winkler M, Pohlmann S. Virion Background and Efficiency of Virion Incorporation Determine Susceptibility of Simian Immunodeficiency Virus Env-Driven Viral Entry to Inhibition by IFITM Proteins. J Virol. 2017;91(2). doi: 10.1128/JVI.01488-16 . 27807233 10.1128/JVI.01488-16 PMC5215347

[32] Weidner JM, Jiang D, Pan XB, Chang J, Block TM, Guo JT. Interferon-induced cell membrane proteins, IFITM3 and tetherin, inhibit vesicular stomatitis virus infection via distinct mechanisms. J Virol. 2010;84(24):12646–57. doi: 10.1128/JVI.01328-10 . 20943977 10.1128/JVI.01328-10 PMC3004348

[33] Warren CJ, Griffin LM, Little AS, Huang IC, Farzan M, Pyeon D. The antiviral restriction factors IFITM1, 2 and 3 do not inhibit infection of human papillomavirus, cytomegalovirus and adenovirus. PLoS One. 2014;9(5):e96579 Epub 2014/05/16. doi: 10.1371/journal.pone.0096579 . 24827144 10.1371/journal.pone.0096579 PMC4020762

[34] Lin TY, Chin CR, Everitt AR, Clare S, Perreira JM, Savidis G, et al Amphotericin B increases influenza A virus infection by preventing IFITM3-mediated restriction. Cell Rep. 2013;5(4):895–908. Epub 2013/11/26. doi: 10.1016/j.celrep.2013.10.033 . 24268777 10.1016/j.celrep.2013.10.033 PMC3898084

[35] Li K, Markosyan RM, Zheng YM, Golfetto O, Bungart B, Li M, et al IFITM proteins restrict viral membrane hemifusion. PLoS Pathog. 2013;9(1):e1003124 Epub 2013/01/30. doi: 10.1371/journal.ppat.1003124 . 23358889 10.1371/journal.ppat.1003124 PMC3554583

[36] Jia R, Xu F, Qian J, Yao Y, Miao C, Zheng YM, et al Identification of an endocytic signal essential for the antiviral action of IFITM3. Cell Microbiol. 2014 Epub 2014/02/14. doi: 10.1111/cmi.12262 . 24521078 10.1111/cmi.12262 PMC4065222

[37] Chesarino NM, McMichael TM, Hach JC, Yount JS. Phosphorylation of the Antiviral Protein IFITM3 Dually Regulates its Endocytosis and Ubiquitination. J Biol Chem. 2014 Epub 2014/03/15. doi: 10.1074/jbc.M114.557694 . 24627473 10.1074/jbc.M114.557694 PMC4002105

[38] Gerlach T, Hensen L, Matrosovich T, Bergmann J, Winkler M, Peteranderl C, et al pH-optimum of hemagglutinin-mediated membrane fusion determines sensitivity of influenza A viruses to the interferon-induced antiviral state and IFITMs. J Virol. 2017 doi: 10.1128/JVI.00246-17 . 28356532 10.1128/JVI.00246-17 PMC5432869

[39] Desai TM, Marin M, Mason C, Melikyan GB. pH regulation in early endosomes and interferon-inducible transmembrane proteins control avian retrovirus fusion. J Biol Chem. 2017 doi: 10.1074/jbc.M117.783878 . 28341742 10.1074/jbc.M117.783878 PMC5427263

[40] Plemper RK. Cell entry of enveloped viruses. Curr Opin Virol. 2011;1(2):92–100. Epub 2011/09/20. doi: 10.1016/j.coviro.2011.06.002 . 21927634 10.1016/j.coviro.2011.06.002 PMC3171968

[41] Baquero E, Albertini AA, Vachette P, Lepault J, Bressanelli S, Gaudin Y. Intermediate conformations during viral fusion glycoprotein structural transition. Curr Opin Virol. 2013;3(2):143–50. Epub 2013/04/09. doi: 10.1016/j.coviro.2013.03.006 . 23562213 10.1016/j.coviro.2013.03.006 PMC7172239

[42] Miyauchi K, Kim Y, Latinovic O, Morozov V, Melikyan GB. HIV enters cells via endocytosis and dynamin-dependent fusion with endosomes. Cell. 2009;137(3):433–44. Epub 2009/05/05. doi: 10.1016/j.cell.2009.02.046 . 19410541 10.1016/j.cell.2009.02.046 PMC2696170

[43] Desai TM, Marin M, Chin CR, Savidis G, Brass AL, Melikyan GB. IFITM3 restricts influenza A virus entry by blocking the formation of fusion pores following virus-endosome hemifusion. PLoS Pathog. 2014;10(4):e1004048 Epub 2014/04/05. doi: 10.1371/journal.ppat.1004048 . 24699674 10.1371/journal.ppat.1004048 PMC3974867

[44] Amini-Bavil-Olyaee S, Choi YJ, Lee JH, Shi M, Huang IC, Farzan M, et al The antiviral effector IFITM3 disrupts intracellular cholesterol homeostasis to block viral entry. Cell Host Microbe. 2013;13(4):452–64. Epub 2013/04/23. doi: 10.1016/j.chom.2013.03.006 . 23601107 10.1016/j.chom.2013.03.006 PMC3646482

[45] Wrensch F, Winkler M, Pohlmann S. IFITM proteins inhibit entry driven by the MERS-coronavirus spike protein: evidence for cholesterol-independent mechanisms. Viruses. 2014;6(9):3683–98. doi: 10.3390/v6093683 . 25256397 10.3390/v6093683 PMC4189045

[46] Fu B, Wang L, Li S, Dorf ME. ZMPSTE24 defends against influenza and other pathogenic viruses. J Exp Med. 2017 doi: 10.1084/jem.20161270 . 28246125 10.1084/jem.20161270 PMC5379977

[47] Wang Y, Pan Q, Ding S, Wang Z, Yu J, Finzi A, et al The V3 Loop of HIV-1 Env Determines Viral Susceptibility to IFITM3 Impairment of Viral Infectivity. J Virol. 2017;91(7). doi: 10.1128/JVI.02441-16 . 28100616 10.1128/JVI.02441-16 PMC5355610

[48] Wu WL, Grotefend CR, Tsai MT, Wang YL, Radic V, Eoh H, et al Delta20 IFITM2 differentially restricts X4 and R5 HIV-1. Proc Natl Acad Sci U S A. 2017;114(27):7112–7. doi: 10.1073/pnas.1619640114 . 28630320 10.1073/pnas.1619640114 PMC5502592

[49] Gruffat H, Manet E, Sergeant A. MEF2-mediated recruitment of class II HDAC at the EBV immediate early gene BZLF1 links latency and chromatin remodeling. EMBO Rep. 2002;3(2):141–6. doi: 10.1093/embo-reports/kvf031 . 11818339 10.1093/embo-reports/kvf031 PMC1083972

[50] Glaser R, Zhang HY, Yao KT, Zhu HC, Wang FX, Li GY, et al Two epithelial tumor cell lines (HNE-1 and HONE-1) latently infected with Epstein-Barr virus that were derived from nasopharyngeal carcinomas. Proc Natl Acad Sci U S A. 1989;86(23):9524–8. . 2556716 10.1073/pnas.86.23.9524 PMC298529

[51] Strobel B, Miller FD, Rist W, Lamla T. Comparative Analysis of Cesium Chloride- and Iodixanol-Based Purification of Recombinant Adeno-Associated Viral Vectors for Preclinical Applications. Hum Gene Ther Methods. 2015;26(4):147–57. doi: 10.1089/hgtb.2015.051 . 26222983 10.1089/hgtb.2015.051 PMC4554548

[52] Dreux M, Garaigorta U, Boyd B, Decembre E, Chung J, Whitten-Bauer C, et al Short-range exosomal transfer of viral RNA from infected cells to plasmacytoid dendritic cells triggers innate immunity. Cell Host Microbe. 2012;12(4):558–70. doi: 10.1016/j.chom.2012.08.010 . 23084922 10.1016/j.chom.2012.08.010 PMC3479672

[53] Compton AA, Roy N, Porrot F, Billet A, Casartelli N, Yount JS, et al Natural mutations in IFITM3 modulate post-translational regulation and toggle antiviral specificity. EMBO Rep. 2016;17(11):1657–71. doi: 10.15252/embr.201642771 . 27601221 10.15252/embr.201642771 PMC5090704

[54] Xie M, Xuan B, Shan J, Pan D, Sun Y, Shan Z, et al Human cytomegalovirus exploits interferon-induced transmembrane proteins to facilitate morphogenesis of the virion assembly compartment. J Virol. 2015;89(6):3049–61. doi: 10.1128/JVI.03416-14 . 25552713 10.1128/JVI.03416-14 PMC4337551

[55] Zhu X, He Z, Yuan J, Wen W, Huang X, Hu Y, et al IFITM3-containing exosome as a novel mediator for anti-viral response in dengue virus infection. Cell Microbiol. 2014 Epub 2014/08/19. doi: 10.1111/cmi.12339 . 25131332 10.1111/cmi.12339 PMC7162390

[56] Rhee SS, Hunter E. A single amino acid substitution within the matrix protein of a type D retrovirus converts its morphogenesis to that of a type C retrovirus. Cell. 1990;63(1):77–86. . 2170021 10.1016/0092-8674(90)90289-q

[57] Garrus JE, von Schwedler UK, Pornillos OW, Morham SG, Zavitz KH, Wang HE, et al Tsg101 and the vacuolar protein sorting pathway are essential for HIV-1 budding. Cell. 2001;107(1):55–65. . 11595185 10.1016/s0092-8674(01)00506-2

[58] Langelier C, von Schwedler UK, Fisher RD, De Domenico I, White PL, Hill CP, et al Human ESCRT-II complex and its role in human immunodeficiency virus type 1 release. J Virol. 2006;80(19):9465–80. doi: 10.1128/JVI.01049-06 . 16973552 10.1128/JVI.01049-06 PMC1617254

[59] Ott DE. Purification of HIV-1 virions by subtilisin digestion or CD45 immunoaffinity depletion for biochemical studies. Methods Mol Biol. 2009;485:15–25. Epub 2008/11/21. doi: 10.1007/978-1-59745-170-3_2 . 19020815 10.1007/978-1-59745-170-3_2

[60] Bartosch B, Cosset FL. Studying HCV cell entry with HCV pseudoparticles (HCVpp). Methods Mol Biol. 2009;510:279–93. doi: 10.1007/978-1-59745-394-3_21 . 19009269 10.1007/978-1-59745-394-3_21

[61] Keele BF, Giorgi EE, Salazar-Gonzalez JF, Decker JM, Pham KT, Salazar MG, et al Identification and characterization of transmitted and early founder virus envelopes in primary HIV-1 infection. Proc Natl Acad Sci U S A. 2008;105(21):7552–7. doi: 10.1073/pnas.0802203105 . 18490657 10.1073/pnas.0802203105 PMC2387184

[62] Zhao X, Guo F, Liu F, Cuconati A, Chang J, Block TM, et al Interferon induction of IFITM proteins promotes infection by human coronavirus OC43. Proc Natl Acad Sci U S A. 2014 doi: 10.1073/pnas.1320856111 . 24753610 10.1073/pnas.1320856111 PMC4020042

[63] Yount JS, Moltedo B, Yang YY, Charron G, Moran TM, Lopez CB, et al Palmitoylome profiling reveals S-palmitoylation-dependent antiviral activity of IFITM3. Nat Chem Biol. 2010;6(8):610–4. Epub 2010/07/06. doi: 10.1038/nchembio.405 . 20601941 10.1038/nchembio.405 PMC2928251

[64] Ding S, Pan Q, Liu SL, Liang C. HIV-1 mutates to evade IFITM1 restriction. Virology. 2014;454–455:11–24. Epub 2014/04/15. doi: 10.1016/j.virol.2014.01.020 . 24725927 10.1016/j.virol.2014.01.020 PMC4274668

[65] Zhong J, Gastaminza P, Chung J, Stamataki Z, Isogawa M, Cheng G, et al Persistent hepatitis C virus infection in vitro: coevolution of virus and host. J Virol. 2006;80(22):11082–93. doi: 10.1128/JVI.01307-06 . 16956932 10.1128/JVI.01307-06 PMC1642175

[66] Tatsuo H, Ono N, Tanaka K, Yanagi Y. [The cellular receptor for measles virus: SLAM (CDw 150)]. Uirusu. 2000;50(2):289–96. . 11276818

[67] Clavel F, Charneau P. Fusion from without directed by human immunodeficiency virus particles. J Virol. 1994;68(2):1179–85. . 8289347 10.1128/jvi.68.2.1179-1185.1994 PMC236557

[68] Berlioz C, Darlix JL. An internal ribosomal entry mechanism promotes translation of murine leukemia virus gag polyprotein precursors. J Virol. 1995;69(4):2214–22. . 7884868 10.1128/jvi.69.4.2214-2222.1995 PMC188890

[69] Giraudon P, Jacquier MF, Wild TF. Antigenic analysis of African measles virus field isolates: identification and localisation of one conserved and two variable epitope sites on the NP protein. Virus Res. 1988;10(2–3):137–52. . 2457995 10.1016/0168-1702(88)90011-1

[70] Reynard O, Borowiak M, Volchkova VA, Delpeut S, Mateo M, Volchkov VE. Ebolavirus glycoprotein GP masks both its own epitopes and the presence of cellular surface proteins. J Virol. 2009;83(18):9596–601. doi: 10.1128/JVI.00784-09 . 19587051 10.1128/JVI.00784-09 PMC2738224

[71] Wobus CE, Hugle-Dorr B, Girod A, Petersen G, Hallek M, Kleinschmidt JA. Monoclonal antibodies against the adeno-associated virus type 2 (AAV-2) capsid: epitope mapping and identification of capsid domains involved in AAV-2-cell interaction and neutralization of AAV-2 infection. J Virol. 2000;74(19):9281–93. . 10982375 10.1128/jvi.74.19.9281-9293.2000 PMC102127

[72] Bartosch B, Dubuisson J, Cosset FL. Infectious hepatitis C virus pseudo-particles containing functional E1-E2 envelope protein complexes. J Exp Med. 2003;197(5):633–42. doi: 10.1084/jem.20021756 . 12615904 10.1084/jem.20021756 PMC2193821

[73] Follenzi A, Naldini L. Generation of HIV-1 derived lentiviral vectors. Methods Enzymol. 2002;346:454–65. . 11883085 10.1016/s0076-6879(02)46071-5

[74] Mangeot PE, Duperrier K, Negre D, Boson B, Rigal D, Cosset FL, et al High levels of transduction of human dendritic cells with optimized SIV vectors. Mol Ther. 2002;5(3):283–90. doi: 10.1006/mthe.2002.0541 . 11863418 10.1006/mthe.2002.0541

[75] Jarrosson-Wuilleme L, Goujon C, Bernaud J, Rigal D, Darlix JL, Cimarelli A. Transduction of nondividing human macrophages with gammaretrovirus-derived vectors. J Virol. 2006;80(3):1152–9. doi: 10.1128/JVI.80.3.1152-1159.2006 . 16414992 10.1128/JVI.80.3.1152-1159.2006 PMC1346929

[76] Wang GZ, Goff SP. Postentry restriction of Mason-Pfizer monkey virus in mouse cells. J Virol. 2015;89(5):2813–9. doi: 10.1128/JVI.03051-14 . 25540373 10.1128/JVI.03051-14 PMC4325713

[77] Grimm D, Kay MA, Kleinschmidt JA. Helper virus-free, optically controllable, and two-plasmid-based production of adeno-associated virus vectors of serotypes 1 to 6. Mol Ther. 2003;7(6):839–50. . 12788658 10.1016/s1525-0016(03)00095-9

[78] Pierson TC, Diamond MS, Ahmed AA, Valentine LE, Davis CW, Samuel MA, et al An infectious West Nile virus that expresses a GFP reporter gene. Virology. 2005;334(1):28–40. doi: 10.1016/j.virol.2005.01.021 . 15749120 10.1016/j.virol.2005.01.021

[79] Ostertag D, Hoblitzell-Ostertag TM, Perrault J. Cell-type-specific growth restriction of vesicular stomatitis virus polR mutants is linked to defective viral polymerase function. J Virol. 2007;81(2):492–502. doi: 10.1128/JVI.01217-06 . 17065214 10.1128/JVI.01217-06 PMC1797469

[80] Devaux P, Cattaneo R. Measles virus phosphoprotein gene products: conformational flexibility of the P/V protein amino-terminal domain and C protein infectivity factor function. J Virol. 2004;78(21):11632–40. doi: 10.1128/JVI.78.21.11632-11640.2004 . 15479804 10.1128/JVI.78.21.11632-11640.2004 PMC523285

[81] Martinez MJ, Biedenkopf N, Volchkova V, Hartlieb B, Alazard-Dany N, Reynard O, et al Role of Ebola virus VP30 in transcription reinitiation. J Virol. 2008;82(24):12569–73. doi: 10.1128/JVI.01395-08 . 18829754 10.1128/JVI.01395-08 PMC2593317

[82] Kato T, Date T, Miyamoto M, Furusaka A, Tokushige K, Mizokami M, et al Efficient replication of the genotype 2a hepatitis C virus subgenomic replicon. Gastroenterology. 2003;125(6):1808–17. . 14724833 10.1053/j.gastro.2003.09.023

[83] Bridgen A, Dalrymple DA, Elliott RM. Dugbe nairovirus S segment: correction of published sequence and comparison of five isolates. Virology. 2002;294(2):364–71. doi: 10.1006/viro.2001.1324 . 12009878 10.1006/viro.2001.1324

[84] Caplen H, Peters CJ, Bishop DH. Mutagen-directed attenuation of Rift Valley fever virus as a method for vaccine development. J Gen Virol. 1985;66 (Pt 10):2271–7. doi: 10.1099/0022-1317-66-10-2271 . 4045430 10.1099/0022-1317-66-10-2271

[85] Wulff H, McIntosh BM, Hamner DB, Johnson KM. Isolation of an arenavirus closely related to Lassa virus from Mastomys natalensis in south-east Africa. Bull World Health Organ. 1977;55(4):441–4. . 304387 PMC2366678

[86] Freed EO, Englund G, Martin MA. Role of the basic domain of human immunodeficiency virus type 1 matrix in macrophage infection. J Virol. 1995;69(6):3949–54. . 7745752 10.1128/jvi.69.6.3949-3954.1995 PMC189124

[87] Hanika A, Larisch B, Steinmann E, Schwegmann-Wessels C, Herrler G, Zimmer G. Use of influenza C virus glycoprotein HEF for generation of vesicular stomatitis virus pseudotypes. J Gen Virol. 2005;86(Pt 5):1455–65. doi: 10.1099/vir.0.80788-0 . 15831958 10.1099/vir.0.80788-0

[88] Goujon C, Jarrosson-Wuilleme L, Bernaud J, Rigal D, Darlix JL, Cimarelli A. With a little help from a friend: increasing HIV transduction of monocyte-derived dendritic cells with virion-like particles of SIV(MAC). Gene Ther. 2006;13(12):991–4. doi: 10.1038/sj.gt.3302753 . 16525481 10.1038/sj.gt.3302753

[89] Goujon C, Riviere L, Jarrosson-Wuilleme L, Bernaud J, Rigal D, Darlix JL, et al SIVSM/HIV-2 Vpx proteins promote retroviral escape from a proteasome-dependent restriction pathway present in human dendritic cells. Retrovirology. 2007;4:2 doi: 10.1186/1742-4690-4-2 . 17212817 10.1186/1742-4690-4-2 PMC1779362

[90] Berger G, Durand S, Goujon C, Nguyen XN, Cordeil S, Darlix JL, et al A simple, versatile and efficient method to genetically modify human monocyte-derived dendritic cells with HIV-1-derived lentiviral vectors. Nat Protoc. 2011;6(6):806–16. doi: 10.1038/nprot.2011.327 . 21637200 10.1038/nprot.2011.327

[91] Laguette N, Sobhian B, Casartelli N, Ringeard M, Chable-Bessia C, Segeral E, et al SAMHD1 is the dendritic- and myeloid-cell-specific HIV-1 restriction factor counteracted by Vpx. Nature. 2011;474(7353):654–7. doi: 10.1038/nature10117 . 21613998 10.1038/nature10117 PMC3595993

[92] Hrecka K, Hao C, Gierszewska M, Swanson SK, Kesik-Brodacka M, Srivastava S, et al Vpx relieves inhibition of HIV-1 infection of macrophages mediated by the SAMHD1 protein. Nature. 2011;474(7353):658–61. doi: 10.1038/nature10195 . 21720370 10.1038/nature10195 PMC3179858

