# Supplementary material for: Interference with the production of infectious viral particles and bimodal inhibition of replication are broadly conserved antiviral properties of IFITMs
Source: PLoS Pathog. 2017 Sep 28;13(9):e1006610. doi: 10.1371/journal.ppat.1006610 (PMC5619827; doi:10.1371/journal.ppat.1006610)
Supplement: S1 Table — (DOCX) [file ppat.1006610.s001.docx]

**Supplementary Table S1. Viruses and viral systems used in this study**

| **Virus** | **viral Strain/ clone** | **Reference** |
| --- | --- | --- |
| Human Immunodeficiency type 1  (HIV-1) | NL4-3-based vector | 73 |
| Simian immunodeficiency  (SIV_MAC_) | SIV_MAC239_-based vector | 74 |
| Murine Leukemia  (MLV) | Moloney-MLV based vector | 75 |
| Mason-Pfizer Monkey  (MPMV) | MPMV-based vector | 76 |
| Vesicular Stomatitis  (VSV) | Indiana serotype | 79 |
| Vesicular Stomatitis pseudotypes  (VSVpp) | Indiana serotype | 87 |
| Measles  (MeV) | vaccine strain, Moraten genome | 80 |
| Ebola  (EBOV) | Zaire | 81 |
| West Nile  (WNV) | pWNII-GFP (strain 956 D117 3B, lineage II) | 78 |
| Hepatitis C  (HCV) | JFH-1 | 82 |
| Dugbe  (DUGV) | isolate IbH 11480 | 83 |
| Rift Valley Fever  (RVFV) | MP-12 | 84 |
| Mopeia  (MOPV) | strain AN21366 | 85 |
| Epstein-Barr  (EBV) | Akata strain | 50 |
| Adeno-Associated Virus  (AAV) | AAV2-based vector | 77 |
| Additional HIV-1 strains | NL-AD8; WITO; CH40; CH106 | 86,61 |
