# Supplementary material for: Interference with the production of infectious viral particles and bimodal inhibition of replication are broadly conserved antiviral properties of IFITMs
Source: PLoS Pathog. 2017 Sep 28;13(9):e1006610. doi: 10.1371/journal.ppat.1006610 (PMC5619827; doi:10.1371/journal.ppat.1006610)
Supplement: S2 Table — (PDF) [file ppat.1006610.s002.pdf]

Supplementary Table S2. List of antibodies and oligonucleotides used in this study

VIRUS	PCR OLIGONUCLEOTIDES FORWARD/REVERSE (WHEN APPLICABLE)	ANTIBODIES (REFERENCE)
Human Immunodeficiency type 1 (HIV-1)	-	anti-Gag/p24, clone 183-H5C from the AIDS Reagents Program of the NIH
Simian immunodeficiency (SIV_{MAC})	-	anti-Gag/p24, clone 183-H5C from the AIDS Reagents Program of the NIH
Murine Leukemia (MLV)	-	anti-MLV Capsid (68)
Mason-Pfizer Monkey (MPMV)	-	*NA*
Vesicular Stomatitis (VSV)	GAACGGCATCAAGGTGAACT-TGCTCAGGTAGTGGTTGTCG	anti-VSVG, clone P5D4, Sigma
Measles (MeV)	GCAGGGCAATCTCACAATCAGG-GCTGACCTTCGACTGTCCT	anti-N (69)
Ebola (EBOV)	CGGAGGCTTTAACCAATA-TCATACATGGGAGTGTGGCT	anti-VP40; anti-GP1, -GP2 and -sGP (70)
West Nile (WNV)	CAGTGTCAGACCACACTTTAATGT-CATAGCCAGGGTTATGGCCGCGTGGG	anti-E glycoprotein (4G2, kindly provided by P. Despres)
Hepatitis C (HCV)	TCTGCGGAACCGGTGAGTA-TCAGGCAGTACCACAAGGC	anti-E1 and –E2 (kindly provided by J. McKeating)
Dugbe (DUGV)	CTGGCTCAAGCAGTGGAACT-AGAGGAATTGAGACAAAGTGA	anti-Dugbe NP (kindly provided by IRBA)
Rift Valley Fever (RVFV)	AAAGGAACAATGGACTCTGGTCA-CACTTCTTACTACCATGTCCTCCAAT	anti-N, Gn and NSs for WB and FACS, kindly provided by R. Elliott and M. Flamand (Gn)
Mopeia (MOPV)	CTTTCCCCTGGCGTGTCA-GAATTTTGAAGGCTGCCTTGA<br>Probe-FAM-TTCCCAAAGAACGCTTG	Anti-Z (this study)
Epstein-Barr (EBV)	CGCAGACATGCTCGATGTA-TAGTGGTGCCCCAGGTATG	anti-EB2-homemade; anti-p52/p50 (clone R3, Merck Millipore)
Adeno-Associated (AAV)	ACGACGGCAACTACAAGACC-CTCCTTGAAGTCGATGCCCT	anti-VP1, -VP2, -VP3 (71)
**OTHER ANTIBODIES**		
Anti-tubulin, -actin, -EF1α	Clones: B-5-1-2 (Sigma), 13E5 (Cell Signaling Technology) or AC74 (Sigma), CBP-KK1(Millipore)	
Anti-Flag	Polyclonal-F7425, or monoclonal-F3165 (Sigma); or Abcam-ab72469 for FC	
Anti-IFITM1, 2, 3	Clones : 60074-1-Ig, 12769-1-AP and 11714-1-AP, respectively, Proteintech	

NA, not available for this study; FC, Flow Cytometry
