# Supplementary figures and images for: Interference with the production of infectious viral particles and bimodal inhibition of replication are broadly conserved antiviral properties of IFITMs
Source: PLoS Pathog. 2017 Sep 28;13(9):e1006610. doi: 10.1371/journal.ppat.1006610 (PMC5619827; doi:10.1371/journal.ppat.1006610)

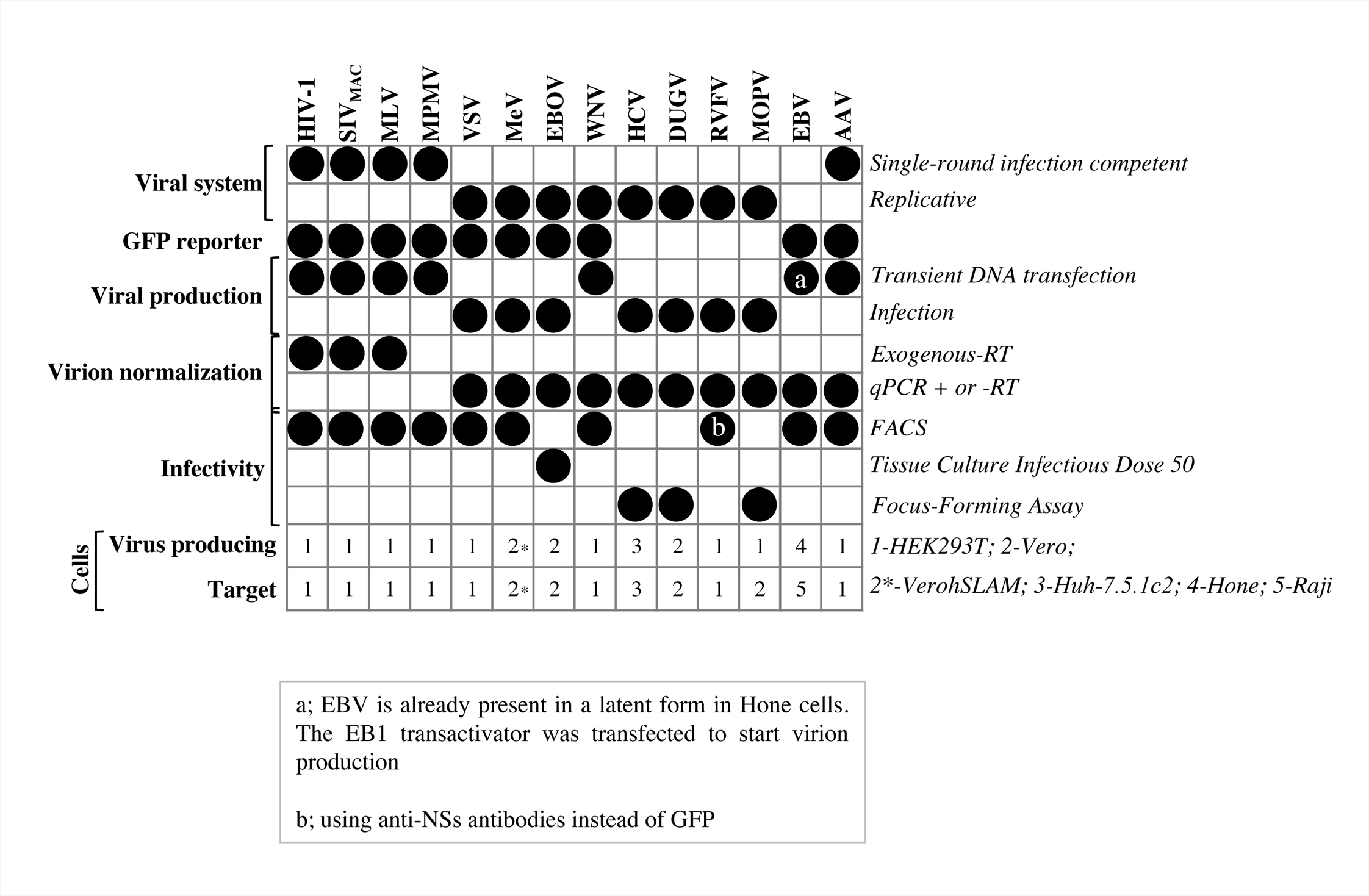

Supplement: S1 Fig — The figure presents schematically the major information on the assays used for each virus to generate the data presented in this study. (TIF) [file ppat.1006610.s003.tif]

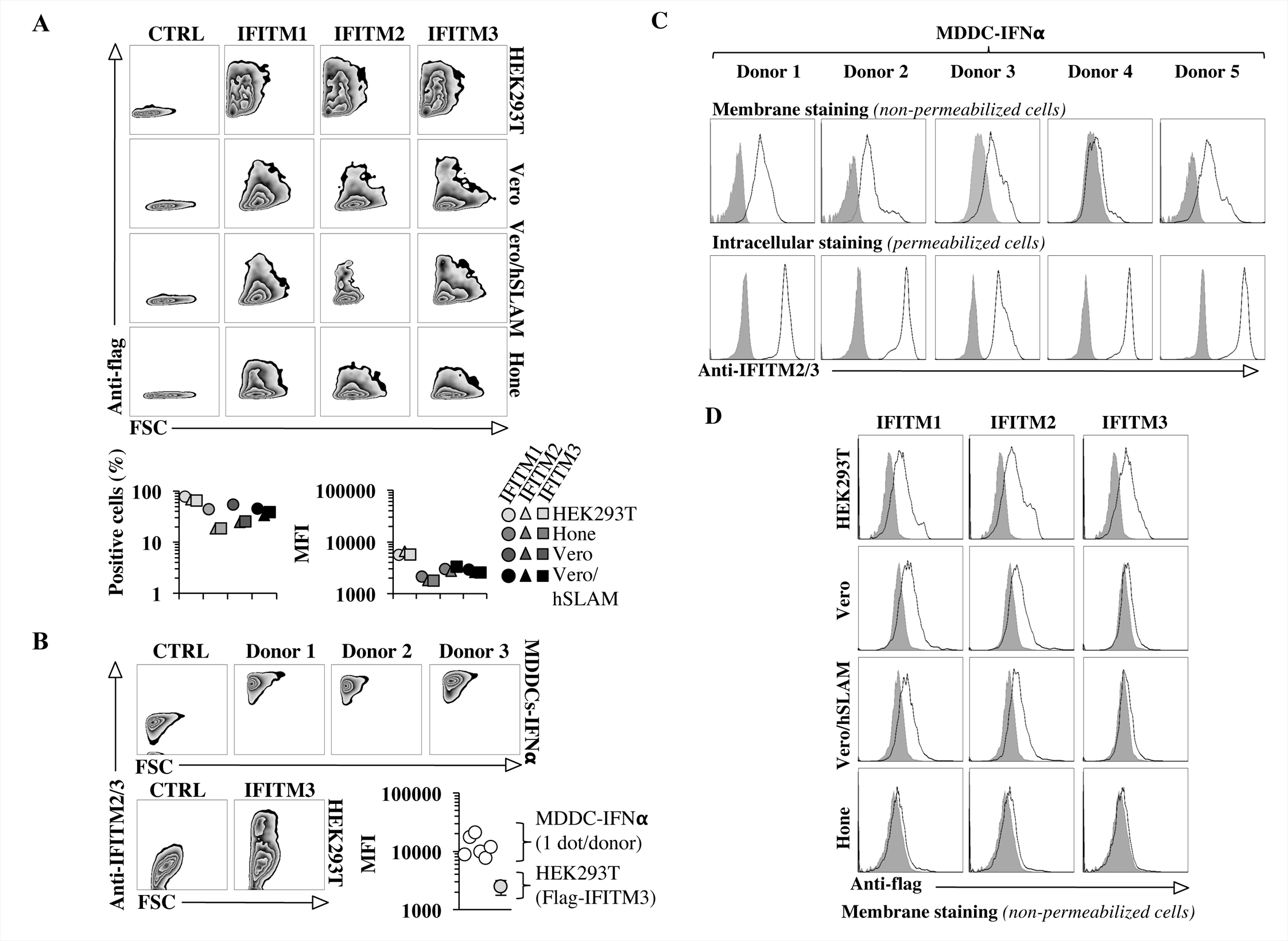

Supplement: S2 Fig — A) To determine the level of Flag-tagged IFITM proteins present upon transient transfection in the different virus-producing cells, cells were analyzed by intracellular staining with an anti-Flag antibody, followed by flow cytometry. Representative panels are shown on top, while quantification of the number of positive cells and their median fluorescence intensity (MFI, that relates to the intracellular levels of IFITM proteins present in positive cells) are shown as graphs. B) The intracellular levels of IFITMs expressed upon transient transfection of the highest expressing cells (HEK293T-transfected with Flag-IFITM3) were compared to the endogenous levels present in monocyte-derived dendritic cells (MDDCs) stimulated for 24 hours with 1.000 U/ml of IFNα, using antibodies directed against IFITM2 and IFITM3. The top panels present typical results obtained in three different donors, while the graph at the bottom compares the MFI obtained (for 6 different donors). Endogenous as well as ectopically-expressed IFITM2/3 proteins are detectably present at the cell surface. C) Monocyte-derived dendritic cells (MDDCs) stimulated for twenty-four hours with IFNα were analyzed by flow cytometry with anti-IFITM2 and IFITM3 antibodies with or without prior cellular permeabilization. IFITM1 is not shown here, because the anti-IFITM1 antibodies we examined did not yield reliable staining in flow cytometry. D) Membrane staining profiles of Flag-tagged IFITM1, 2 and 3 ectopically expressed in the different cell types used in this study. The smaller shift detected in Hone cells is likely due to the lower transfection rate and intracellular accumulation level of IFITMs in this cell line, rather than to a cell type-specific behavior. (TIF) [file ppat.1006610.s004.tif]

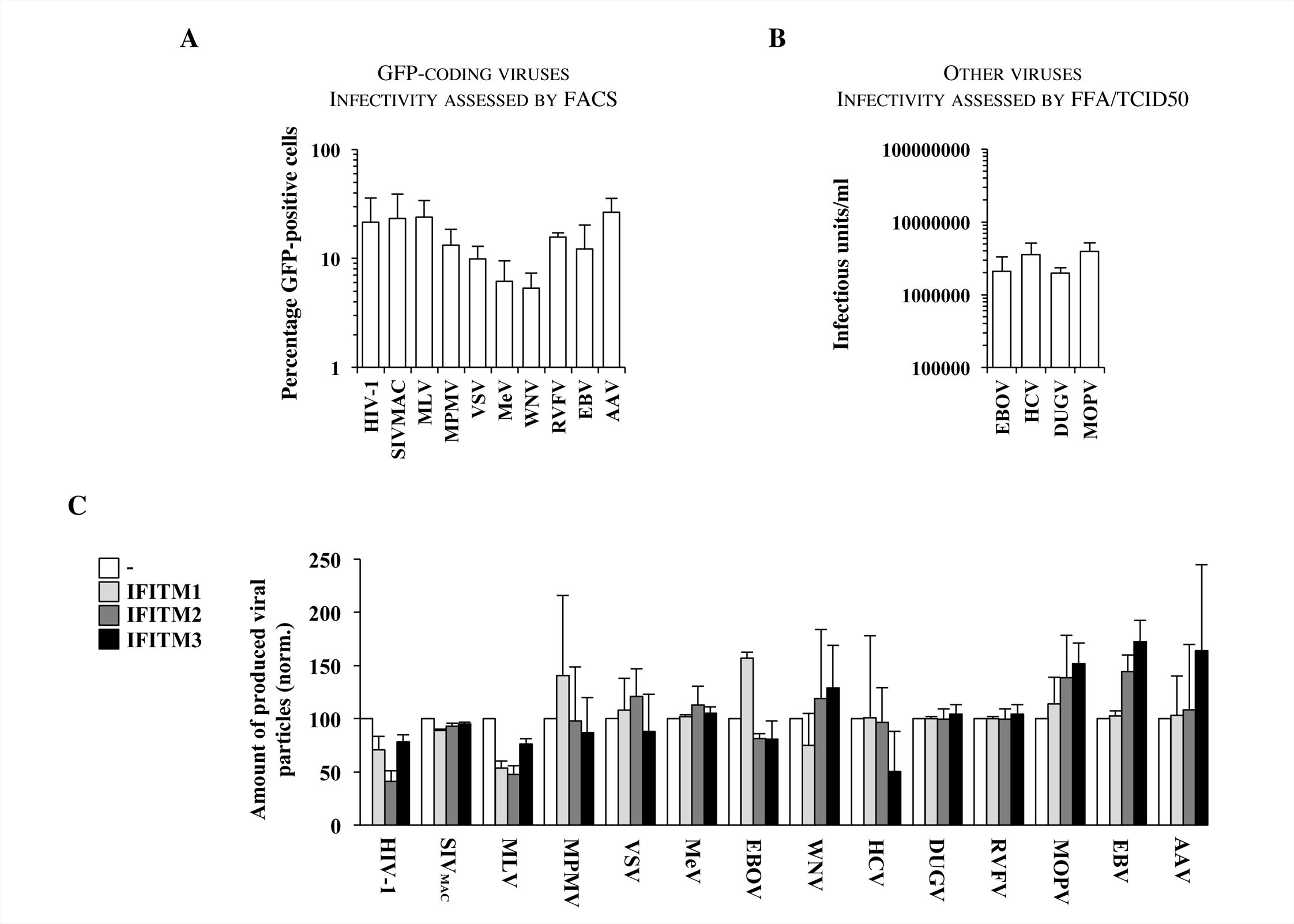

Supplement: S3 Fig — A and B) Comparison of the infectious titers used in this study for the different viruses, as estimated by flow cytometry or FFA/TCID50. The two graphs compare the average infectivity of viral preparations used throughout this study as determined by FACS (in the case of most gfp-bearing viruses), or by FFA/TCID50 (presented here as infectious units/ml). C) Effects of IFITMs on the production of viral particles. The viruses produced in the presence or absence of IFITMs were quantified according to the methods presented in the Supplementary S1 Fig. For each virus, values have been normalized to control virions produced in the absence of IFITMs. The graph presents the averages of 3 to 4 distinct experiments. (TIF) [file ppat.1006610.s005.tif]

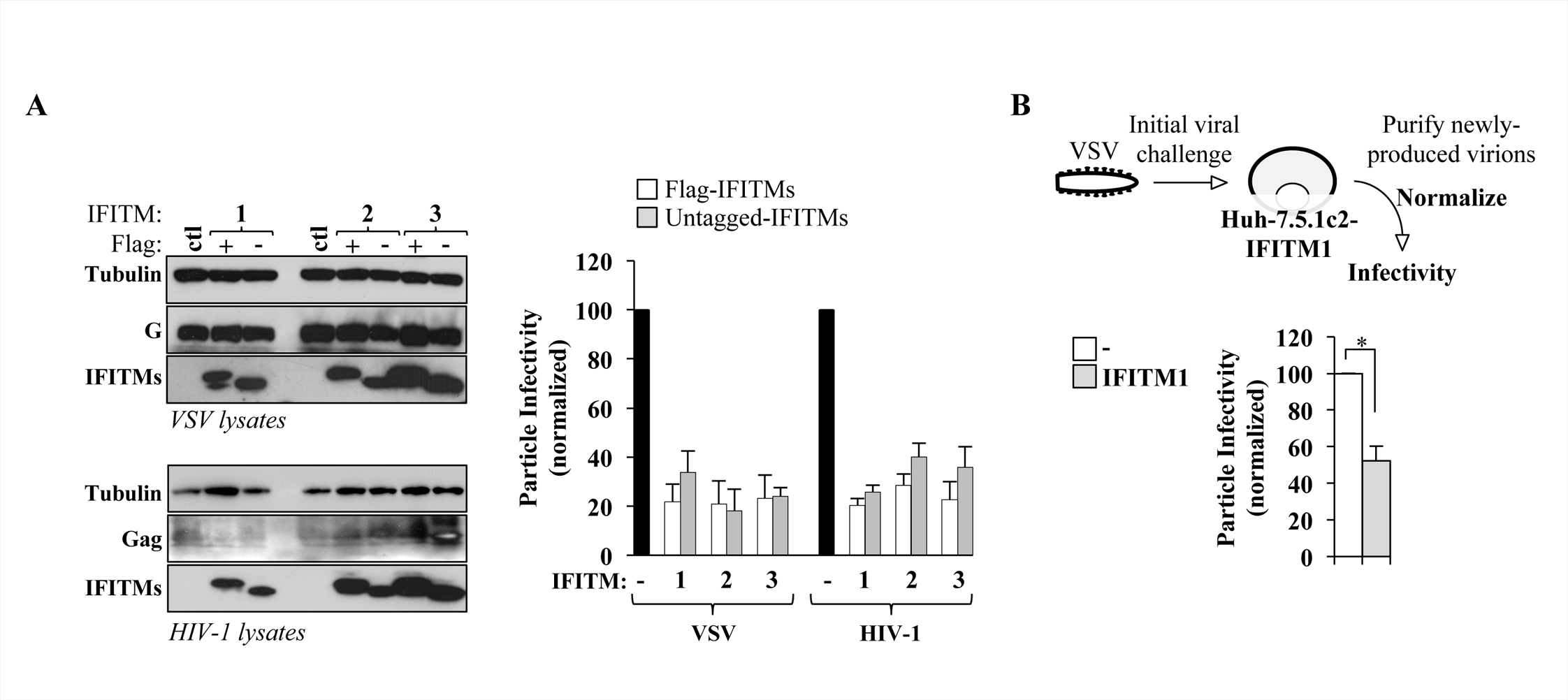

Supplement: S4 Fig — A) Comparison of the antiviral effects exerted by tagged and natural IFITM proteins. HIV-1 and VSV viral particles were produced in cells transfected with DNAs coding Flag-tagged and non-tagged IFITMs. The panels display typical expression patterns of virus-producing cells, while the graph reports averages and SEM obtained from 4 independent experiments. No statistically significant difference was observed between the effects of individual pairs of IFITMs following a Student t test. B) Determination of the antiviral effects of IFITM1 in HuH7 against VSV. To determine whether the lack of antiviral effects observed for HCV virions produced in HuH7 cells in the presence of the different IFITMs presented in Fig 4 was not due to insufficient expression of IFITMs, HuH7 cells expressing IFITM1 (the least expressed among the IFITM members) were used as VSV-producing cells as indicated in the scheme above. Newly-produced viral particles were then normalized and used to challenge HEK293T cells. The graph presents averages and SEM of 3 independent experiments. * = p≤0.05, after a Student t test. (TIF) [file ppat.1006610.s006.tif]

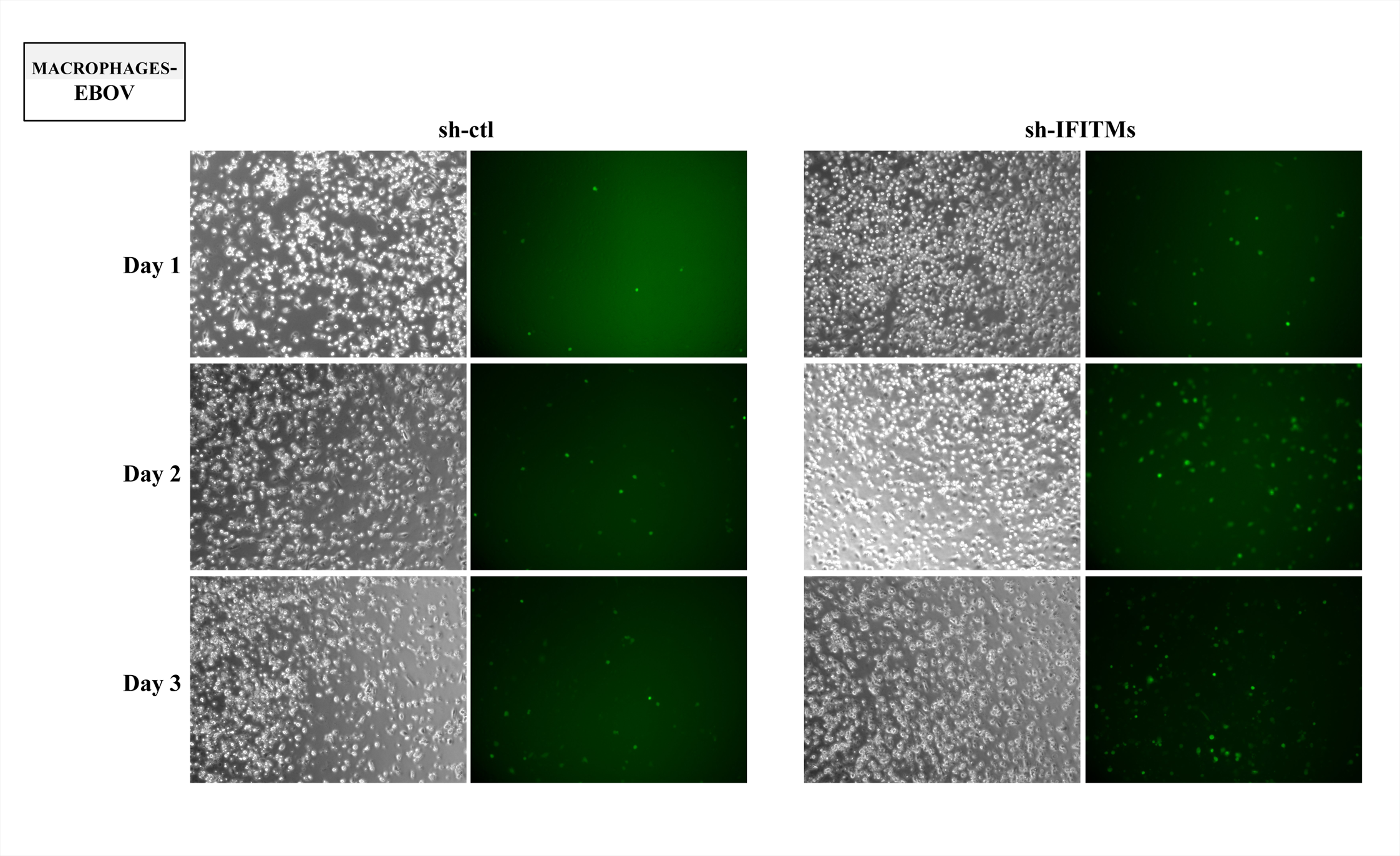

Supplement: S5 Fig — This figure presents the complete analysis of EBOV replication in primary monocyte-derived macrophages (due to space constraints only GFP-positive cells obtained at day 2 are presented in Fig 5). Briefly, blood monocytes were differentiated in macrophages upon incubation with M-CSF during four days in a 24 well-plate format. Cells were then transduced with an MOI of 1 of HIV-1 vectors bearing an shRNA expression cassette directed against control sequences (Luciferase) or against IFITM1, 2 and 3. Four days afterwards, cells were challenged with an MOI of 0.3 of EBOV. Viral spread was analyzed through the accumulation of GFP-positive cells thanks to the virus-coded GFP reporter. (TIF) [file ppat.1006610.s007.tif]

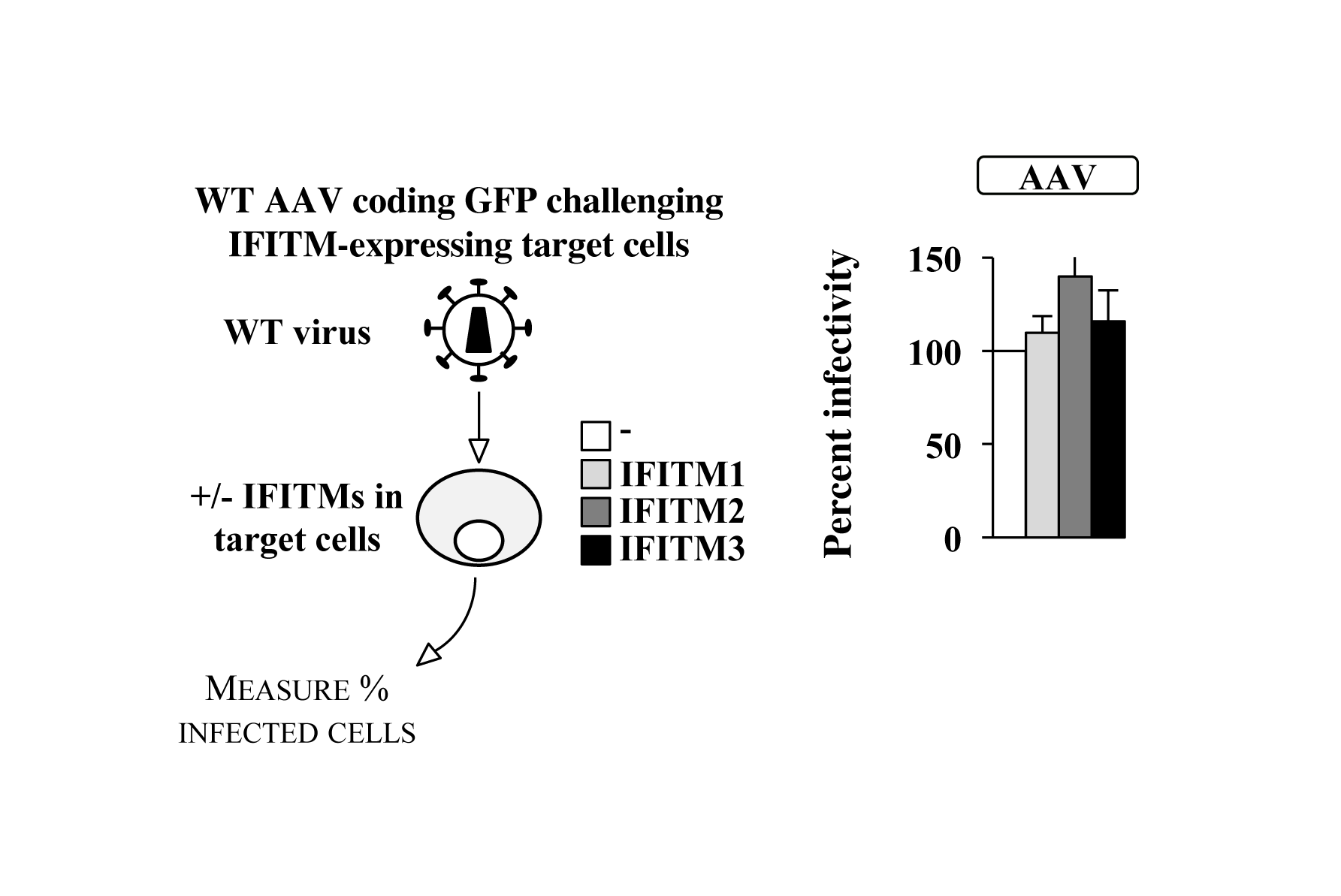

Supplement: S6 Fig — Cells expressing IFITM1, 2 and 3 were challenged with GFP-coding AAV, prior to the quantification of the percentage of GFP-positive cells by flow cytometry. The graph present averages and SEM of 3 independent experiments. (TIF) [file ppat.1006610.s008.tif]
